# In Silico Analysis of Potential Stabilizer Binding Sites at Protein–RNA Interfaces

**DOI:** 10.34133/csbj.0016

**Published:** 2026-03-31

**Authors:** Luis Vollmers, Shu-Yu Chen, Martin Zacharias

**Affiliations:** ^1^ Center for Functional Protein Assemblies, Technical University of Munich, Munich, Germany.; ^2^ Department of Chemistry and Applied Biosciences, ETH Zurich, Zurich, Switzerland.

## Abstract

Stabilizing rather than interfering with protein–protein interactions has emerged as a promising concept for designing drug molecules that can modulate protein–protein interactions. In addition to protein–protein interactions, protein–RNA interactions are involved in numerous cellular metabolic and regulatory processes, and it is desirable to stabilize these interactions, e.g., for potential pharmaceutical applications. In order to assess the potential that lies in protein–RNA interaction modulation by stabilizing drug molecules, we analyzed the binding interfaces of a large set of 87 protein–RNA complexes. Our in silico analysis revealed many interface cavities and potential binding sites that could fit drug-like compounds that can simultaneously interact with the protein and RNA. Through a systematic workflow combining interface pocket detection, molecular docking, and molecular mechanics coupled with the generalized Born surface area (MMGB/SA) calculations, we evaluated multiple pocket detection algorithms and identified key chemical features shared among in silico stabilizer candidates. The docking approach successfully reproduced portions of known experimental ligands and could be valuable for suggesting potential stabilizers of protein–RNA interactions.

## Highlights

• First large-scale in silico investigation of molecular glue druggability in protein–RNA complexes, combining virtual screening, molecular dynamics simulations, and structure prediction models.
• A total of 52,000,000 ligand–receptor complexes were screened, yielding 1,300 candidates for detailed analysis, among which 96 exhibited promising stabilizing potential.
• For 39% of the examined complexes, at least one promising stabilizer candidate was identified in silico.
• Three openly accessible databases were established to support molecular glue discovery, covering both general medicinal compounds and those tailored explicitly to protein–RNA interactions.
• An in-depth analysis of detected protein–RNA interface pockets shows that pocket druggability primarily depends on static structural data.

## Graphical Abstract

**Figure:**
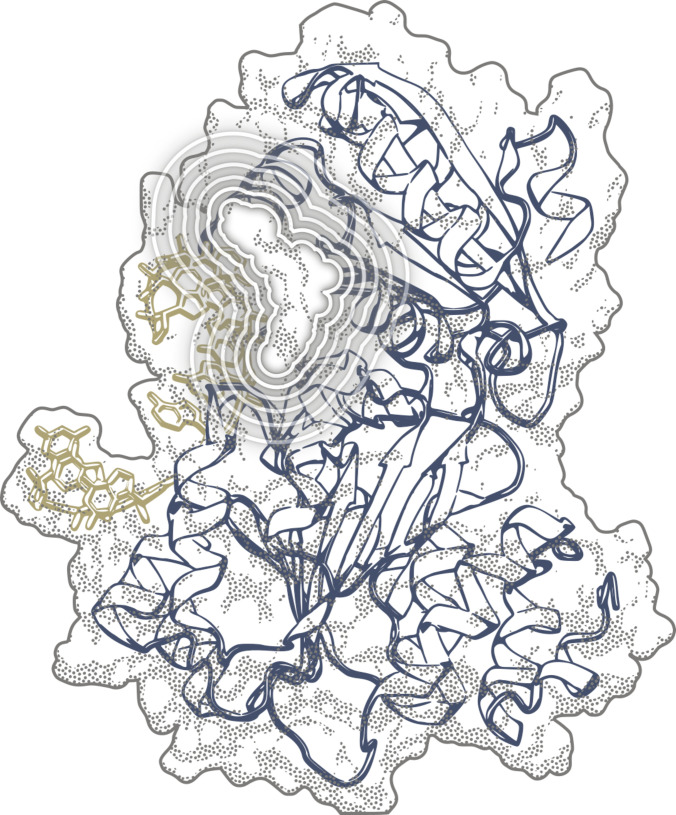

## Introduction

Almost all biological processes are mediated by interactions between proteins and other biomolecules, and modulating these interactions has long been a popular therapeutic strategy [1–5]. While inhibitors against enzymes and protein–protein interactions (PPIs) still dominate small-molecule drug design due to its long history and well-established protocols, stabilization of biomolecule interactions has attracted more and more attention in recent years. It may alleviate the side effects of traditional inhibitors, such as off-target effects resulting from their low selectivity [6–9]. Also, the alveolated interfaces of transient biomolecular complexes offer access to new cavities that may fit drug-like molecules. The pharmacological potential is exemplified by the serendipitous discoveries of cyclosporin A (CsA) [10,11], FK506 [12,13], rapamycin [14,15], and paclitaxel [16–18] in the late 20th century. Despite molecular stabilizers’ therapeutic potential and versatility, they pose a substantial challenge for rational design because they must target multiple biomolecules without inhibiting them.

The introduction of proteolysis-targeting chimeras (PROTACs) in 2001 by Sakamoto et al. [19] offered an approach to rationally bring 2 biomolecules to proximity by introducing a linker between known small ligands specifically binding to each of the partner proteins [20–25]. However, the high molecular weight often causes PROTACs to suffer from membrane permeability and high toxicity. Also, the notorious hook effect due to low cooperativity [26,27] narrows the PROTAC working concentration window [28]. Stabilizers, often termed molecular glues, tend to show high binding cooperativity but their discovery is mostly serendipitous, and the rational design of de novo methods is still in demand [1,8,27,29,30].

In contrast to PPIs, modulation of RNA–protein interactions (RPIs) with small molecules is a much less established field, besides inhibitors such as LIN28 [31,32], HuR [33–35], and YBX1 [36–39]. These drug discovery efforts were driven by classical experimental methodology comprising high-throughput Förster resonance energy transfer, fluorescence anisotropy assays, and surface plasmon resonance spectroscopy [31,34,36]. A main difficulty lies in the large and dynamic interfaces between RNA and proteins, and the higher degradation rate of RNA also complicates the understanding of RNA-binding proteins [40]. Accordingly, there are comparably few reported RPI stabilizers, exemplified by eIF4A-RNA stabilizers rocaglamide A and C5-desmethyl pateamine A [41–43]. Nonetheless, a growing body of research on RPI stabilizers, such as the RNA-PROTAC technique introduced by Ghidini et al. [44] and the photoswitchable molecular glue NCTA introduced by Dohno et al. [45], has shown promise for future development.

It is important to note that drug design targeting RNA molecules has been only marginally successful despite many efforts over the last 30 years. A difficulty in this drug design process is likely due to the limited number and variability of binding cavities in RNA, which are composed of only 4 different nucleotides. The focus on protein–RNA interfaces is expected to provide greater physicochemical and stereochemical variability in interface pockets for specific drug binding than in pure RNA.

In addition to experimental methods, mathematical models are introduced to understand the equilibria of ternary complexes that underpin ligand-mediated protein–RNA stabilization [28,46]. Such computational methods include molecular dynamics (MD) simulations [47–49], in silico pocket detection [49], and molecular docking [49–51]. Recent advances in artificial intelligence (AI), such as Boltz-1 [52], AlphaFold 3 [53], Chai-1 [54], DeepTernary [55], and FKSFold [56], further enable the co-folding of a ternary complex in a single prediction step. However, those machine learning models lack generalizability, e.g., regarding druggable interface pockets, due to the sporadic availability of high-quality 3-dimensional RPI structures.

So far, limited successes in identifying RPI stabilizing compounds and the lack of structural data cast doubt on whether RPIs are suitable targets for drug discovery. Our study aims to address obstacles and dispel doubts by introducing a rigorous computational workflow to expound the druggability of 87 protein–RNA complexes with high-quality structures using interface pocket detection and physics-based analysis. The devised workflow identified numerous complex interface pockets and predicted that many of them could be suitable for drug binding, due to their size and chemical properties. Leveraging a curated database of 34 million compounds, large-scale molecular docking and MD simulations are performed to better understand the protein–RNA interface and the binding of potential stabilizers. In summary, our designed analysis and docking protocols identify protein–RNA interface pockets, estimate relative binding free energies of docked compounds, and also provide practical guidance for future drug discovery efforts in the RPI stabilizer field. Classical and experimental methods, such as high-throughput screening campaigns [57], may benefit from this guidance, particularly from the databases published with this article.

## Methods

**Fig. 1.**
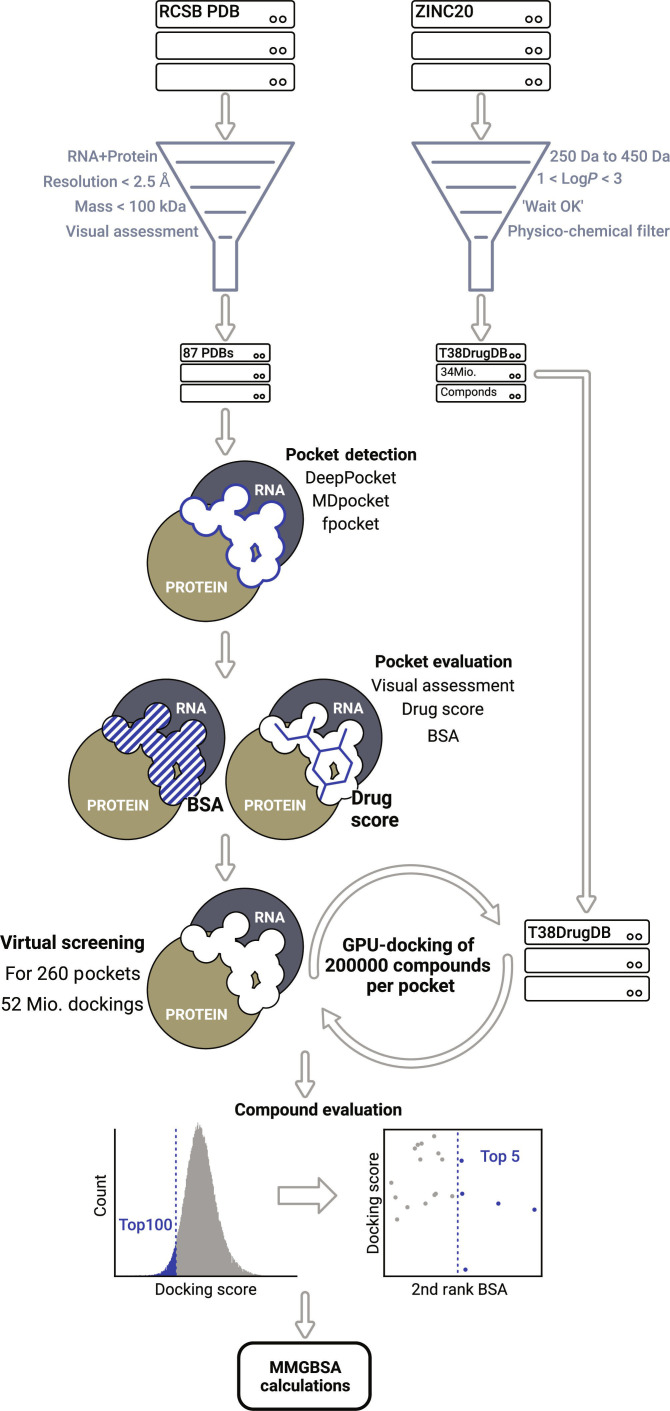
The starting point of the workflow is the PDB website and the ZINC20. From these, custom databases for protein–RNA complexes and small organic compounds are generated. The custom databases contain 87 complexes and 34 million small organic compounds. The physico-chemical filter criteria are listed in Table 1. The protein–RNA complexes are subjected to pocket detection using the fpocket, MDpocket, and DeepPocket programs [73–75]. The most promising interface pocket is then selected for each algorithm. For the resulting 260 pockets, a virtual screening takes place where 200,000 compounds are docked in each pocket, which are then filtered via the docking score [113,114] and the buried surface area. Finally, the 5 most promising docking results for each pocket are simulated and evaluated via MMGB/SA.

Our study employs data curation, docking, and modeling, as well as various postprocessing analyses (see Fig. 1). The data curation entails the chemical compound, complex, and pocket databases. Potent stabilizer candidates are identified via a docking, modeling, and MMGB/SA (molecular mechanics coupled with the generalized Born surface area) workflow. The postprocessing analysis includes experimental validation, machine learning, chemical fragmentation analysis, and structure prediction. All employed methods are computational, and no additional experiments were performed.

**Table 1.** Physicochemical properties to filter the ZINC20 database with. These values represent the averages of the commercially available orally admissible drugs plus/minus their standard deviation [69].

Property	Range (including)
Log*P*	1–3
Mass	250–450 Da
NOCount	2–9
NHOHCount	0–3
TPSA	19–70 A^2^
NumRotatableBonds	1–8
NumAromaticCarbocycles	0–2
NumAromaticHeterocycles	0–1
NumAromaticRings	1–3
FractionCSP3	0.2–0.6

### Screening the RCSB PDB

Firstly, a consistent database of protein–RNA complexes was compiled using the PDB website. The database was queried for entities containing RNA and protein macromolecules via the “advanced search” functionality. A resolution cutoff of 2.5 Å and a molecular weight maximum of 100 kDa were applied to ensure that MD simulations are computationally feasible. This query yielded approximately 500 complexes, which were visually inspected to evaluate the interface quality and size relationships between protein and RNA components. To ensure structural relevance, complexes were retained only if the ratio of RNA to protein atoms fell within the range of 0.1 to 20, the interface comprised a minimum of 25 atoms from each component, and the fraction of interface atoms should be at least 2% to 5% for the protein and RNA chains. Additionally, the maximum spatial extent of the complex was required to be less than 70 Å. Complexes failing to meet these criteria—for example, those in which the RNA engages the protein only marginally, such as 2A8V [58]—were excluded from further investigation. Overly solvent-exposed interfaces, e.g., 5BZ5 [59], were also omitted due to the probable displacement of stabilizer candidates by water molecules. Complex structures derived from nuclear magnetic resonance (NMR) spectroscopy were also omitted because they might not be well represented by a single mean structure, and we plan to investigate structural ensembles in future studies. Ultimately, 87 suitable complexes remained, which are listed in Table S1. From the distilled 87 complexes, 13 have an experimentally resolved binding site that can be leveraged for assessing the in silico result validity (see Table 2).

**Table 2.** PDB entries with experimental ligands at their protein–RNA interfaces. Instead of 2DVI [115], macromolecules from 2DRB [115] were used due to better quality. 2DVI provided the coordinates of cytidinetriphosphate (CTP). Most organic compounds bind preferentially to the protein as cofactors, with 6XKI [43] being a notable exception, in which V6D stabilizes the protein–RNA interface, which the original authors describe as functional inhibition [43]. Complexes containing small organic compounds only bound to one macromolecule were not listed.

PBD-ID	Ligand	Function	Authors	Detected by
2DRB	CTP	Cosubstrate	Tomita et al. [115]	DeepPocket
2HVY	ATP	Buffer	Li and Ye [105]	Fpocket
2ZZM	SAM	Cofactor	Goto-Ito et al. [97]	Not found
3AMT	ATP	Cofactor	Osawa et al. [98]	DeepPocket
3OIN	SAH	Cofactor	Thomas et al. [99]	DeepPocket
4GCW	MES	Buffer	Pellegrini et al. [116]	Not found
4YCP	FMN	Cofactor	Byrne et al. [117]	DeepPocket
5ZQ0	SAH	Cofactor	Jiang et al. [100]	Fpocket
5ZW4	SAM	Cofactor	Ryu et al. [101]	Fpocket
6AAX	SAM	Cofactor	Liu et al. [118]	Fpocket
6XKI	V6D	Stabilizer	Naineni et al. [43]	Fpocket
7K9D	HEPES	Buffer	Yang et al. [119]	Not found
7MJV	SAM	Cofactor	Esakova et al. [102]	DeepPocket

We developed a Python routine using MODELLER [60] and AlphaFold 3 [53] to process the raw PDB files, adding missing atoms and residues and keeping buried metal ions, if applicable. MODELLER was used to account for missing or nonstandard protein residues. For missing RNA residues, the macromolecule was modeled via AlphaFold 3, followed by excision and ligation of the target residue into the original PDB structure with PyMOL [61] to maximize usage of original experimental coordinates. Duplicate RNA and protein chains were excluded unless they directly contacted the interface. The PDB files were used to conduct apo-complex simulations (meaning in the absence of stabilizer compounds) via the Amber MD simulation software [62] at a sodium chloride concentration of 150 mM. After energy minimization, simulations ran for 100 ns at 300 K and 1-bar pressure, with a 4-fs time step enabled by hydrogen mass repartitioning to 3.024 Da. Simulations of the apo-complexes were run to assess complex stability and to generate pocket descriptors related to topological and hydration-specific fluctuations. These pocket properties were analyzed using a custom Python-based framework that leverages MDAnalysis, SciPy, and PyntCloud [63–65]. Water molecules enclosed within the convex hull were counted for 10 equidistant frames of each trajectory per pocket. The absolute water density and relative water density were calculated with the density of bulk water at standard conditions (0.0334 A^−3^). To quantify the structural organization of water molecules within the pocket, the translational order parameter $S_{k}$ is calculated using the distance, $r_{i}$, between a central water oxygen atom and its 4 neighboring water oxygens, and $\bar{r}$, which is the mean of these distances [66,67].

$$S_{k}=\frac{1}{12{\bar{r}}^{2}}\sum_{i}^{4}{\left(r_{i}-\bar{r}\right)}^{2}$$ (1)

These features were generated to train a random forest classifier, as described below (Random forest modeling and feature importance section).

### Generating a database of medicinally relevant compounds

The ZINC20 database contains almost 900 million compounds [68]. Several filters have been applied to distill these compounds to an amount feasible for virtual screening. The filters were designed to match the physicochemical properties of commercially available orally admissible drugs [69]. The built-in query on the ZINC20 web page was used to constrain the molecular weight from 250 to 450 Da and Log*P* values from 1 to 3. The purchasability was set to “wait OK”, and all other filter criteria were applied via a Python script (see Table 1). After removing duplicate entries, approximately 34 million clinically relevant compounds remained. The resulting database, termed T38DrugDB, contains all compounds’ simplified molecular input line entry system (SMILES) strings [70,71], ZINC-ID, PDBQT format, and MOL2 format for querying, docking, and subsequent parameterization with the amberTools software suite [72].

### Pocket detection algorithms

Three pocket detection algorithms were evaluated: fpocket, MDpocket, and DeepPocket [73–75]. Fpocket and DeepPocket are used on the crystallographic PDB files, while MDpocket evaluates 2,500 frames of the apo-complex simulations to factor in pocket dynamics. Fpocket’s maximum alpha sphere radius was increased to 8 Å for better interface pocket detection [73]. For molecular glues, a pocket’s buried surface area (BSA) should be distributed equally between the RNA and protein macromolecules. The smaller of the 2 BSAs was termed second-rank BSA. The BSA is calculated from the solvent-accessible surface area (SASA) obtained from the Shrake–Rupley algorithm implemented in MDTraj [76].

$${\mathrm{BSA}}_{\text{Prot},\text{Pocket}}={\text{SASA}}_{\text{Prot}}+{\text{SASA}}_{\text{Pocket}}-{\text{SASA}}_{\text{Prot},\text{Pocket}}$$ (2)

**Fig. 2.**
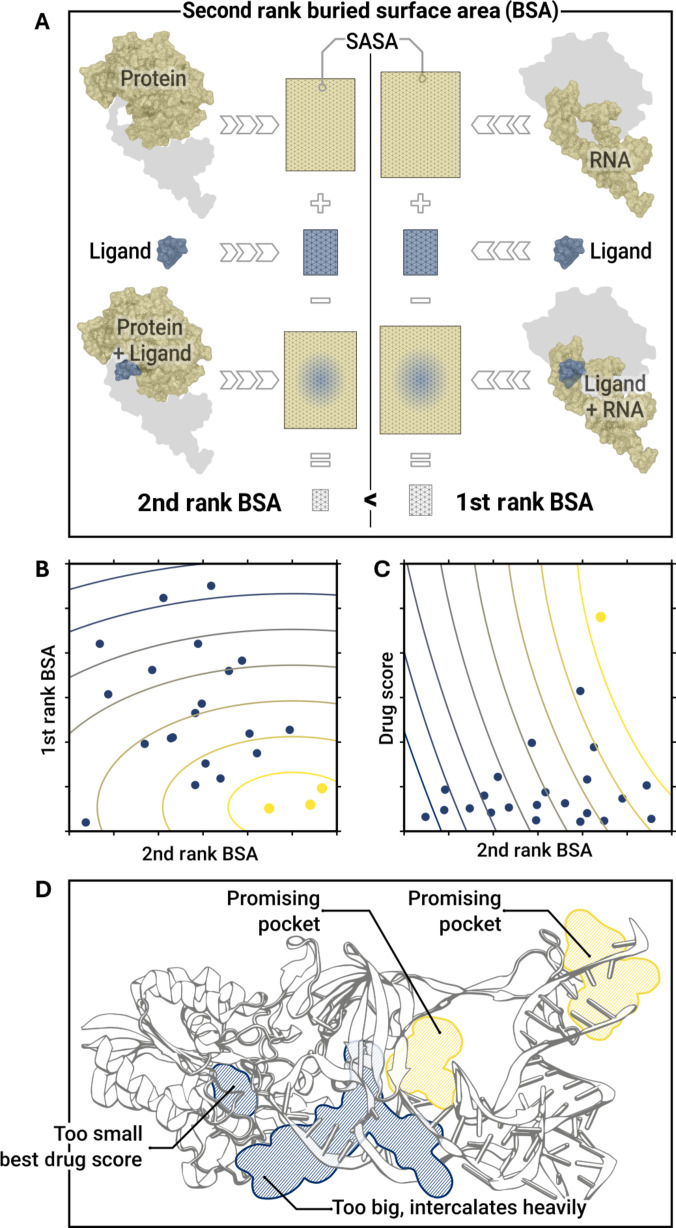
The pocket evaluation process for fpocket and MDpocket. (A) Calculation of BSAs. The SASA of one of the macromolecules (yellow) is calculated and added to the SASA of the pocket or ligand (blue) and then subtracted by the SASA of the pocket–macromolecule combination (blue-yellow gradient). The result is the BSA of the pocket with the corresponding macromolecule. The smaller BSA is an important metric for compound and pocket quality. (B) First-rank BSA pockets plotted against their second-rank BSA. The yellow color indicates promising pockets. (C) Pockets’ drug score plotted against their second-rank BSA. The yellow color indicates promising pockets. (D) Example of visual inspection. The protein–RNA complex (gray ribbons) features 2 promising pockets (yellow) and 2 rejected pockets (blue).

A visual depiction of BSA calculation is shown in Fig. 2A. Selecting high second-rank BSAs ensures profound pocket contacts with the protein and the RNA. Thus, the decision process involved comparing the second-rank BSAs at the protein–RNA interface. Other informative metrics, such as fpocket’s drug score and MDpocket’s apolar alpha-sphere ratio, were also considered. If a clear decision based on these metrics was not possible, the pockets were visually inspected to decide based on expert opinion (see Fig. 2). DeepPocket is an fpocket variant that ranks using a convolutional neural network scoring function. The highest-ranked interface pocket was selected, which MDpocket or fpocket had not already identified. For docking, a cuboid volume was generated from the box dimension with an added margin of 5 Å.

### Virtual screening

A virtual screening was conducted on each identified pocket using a random subsample of 200,000 compounds. In total, 260 pockets were analyzed, resulting in 52 million docking attempts using AutoDock-GPU from the Forli Lab [77]. In the case of fpocket and DeepPocket, the crystal structures were used as docking input. For MDpocket, the simulation frame $t_{\min}$ with the minimum root mean square deviation of atomic positions (RMSD) to the complex’s time-averaged structure was used. Each trajectory snapshot $\vec{x}\left(t_{j}\right)$ is aligned beforehand with the RMSD to the first frame via the rotation matrix $R_{i}^{0}$ and translation vector $T_{i}^{0}$ [78,79].

$$t_{\min}=\text{arg}\;\underset{t_{i}}{\text{min}}\left\|R_{i}^{0}\cdot \vec{x}\left(t_{i}\right)+T_{i}^{0}-\frac{1}{N}\sum_{j}^{N}\left[R_{j}^{0}\cdot \vec{x}\left(t_{j}\right)+T_{j}^{0}\right]\right\|$$ (3)

A newly implemented multistep filtering process identifies in silico stabilizer candidates. First, the 100 compounds with the best docking scores were selected for each pocket. From the top 100 compounds, the 5 compounds with the best second-rank BSA are retained for MMGB/SA calculations [62,72]. The BSA is calculated analogously to Eq. (2) only with the SASA of the ligand instead of the pocket.

### Postprocessing and further analysis

The subsequent analysis of the virtual screening results can be partitioned into 5 main components.

1. Binding affinity calculation and stabilizer candidate identification among each pocket’s top 5 compounds.
2. Assessment of experimental ligands concerning their similarity to our in silico ligands.
3. Random forest classifier prediction of pocket druggability and feature importance analysis.
4. Molecular fragmentation analysis of stabilizer candidates via chemical fingerprints and retrosynthetic bond breaking.
5. Comparison of our in silico ligands to one-step predictions conducted by the Chai-1 model [54].

#### Calculated binding affinities and comparison to experiment

The ligands’ interactions with the macromolecules were evaluated via MMGB/SA [72,80,81]. For each pocket’s top 5 ligands, this analysis yielded the mean interaction free energy to the RNA (∆∆G_RNA_) and mean interaction free energy to the protein (∆∆G_Protein_). For the MMGB/SA calculations, the internal and external dielectric constants are 1 and 80. Effective Born radii are calculated using the GBOBCII model [82]. We defined 3 categories based on the MMGB/SA results. Compounds with ∆∆G_Protein_ and ∆∆G_RNA_ lower than −10 kcal mol^−1^ are labeled elevated stabilizer candidates; compounds with both interaction energies lower than −7 kcal mol^−1^ are labeled basic stabilizer candidates. Any compound above is simply a regular organic ligand.

#### Assessment of experimental ligands

First, the similarities between in silico and experimentally known ligands are highlighted by assessing the overlap between the experimental binding sites and the pocket algorithms’ results. Next, the molecular similarity between the experimental and in silico ligands was assessed using ESPSIM [83]. Precalculated AM1-BCC charges from antechamber version 22.0 and parmchk2 were used to approximate the ligands’ electrostatic potential, as recommended by Bolcato et al. [72,83]. ESPSIM yields 2 similarity scores. One corresponds to the molecular shape, and the other to the electrostatic potential (ESP) similarity. The former assesses the geometrical molecular features, while the latter corresponds to the estimated charge distribution. Since 3-dimensional molecules can have various charge and shape distributions, 100 conformers are generated via RDKit [84] for each of the in silico and experimental ligands. ESPSIM automatically retains the best score. The salience of those similarities and the mean interaction free energy (∆∆G) values from MMGB/SA are discussed.

#### Random forest modeling and feature importance

A random forest classifier is trained on a 77-feature vector to predict the presence of basic and elevated stabilizer candidates within a pocket. This feature vector contains information derived from apo-complex simulations, especially regarding geometrical and pocket hydration properties described in the Screening the RCSB PDB section. Additionally, the top 5 in silico ligands (totaling 1,300 for 260 pockets) were used to generate features relating to the ligands’ BSAs and their docking scores. Scikit-learn’s RandomForestClassifier [85] was used with extensive hyperparameter tuning, nested cross validation (CV), and a superimposed preprocessing grid search handling data imbalance and splitting [86].

Separate models were trained and evaluated for each nested CV fold, resulting in 5 distinct models for each of the 135 preprocessing grid combinations. Model performance was assessed primarily using sensitivity, the receiver operator characteristic (ROC), and the ROC area under the curve (AUC) [87–89]. The trained models were then used to compute the average impurity decrease a feature can achieve on the dataset to estimate the feature’s importance. All analyses were conducted using Python 3 with scikit-learn, imbalanced-learn, NumPy, and pandas [85,90–93]. The complete feature vector and model setup are further detailed in Section S1.

#### Chemical fragmentation analysis

A fragment-based analysis approach was implemented using RDKit [84] and custom Python workflows to explore the chemical space of the compounds and collect key structural features contributing to binding affinity. This analysis is divided into extended connectivity fingerprint (ECFP) and breaking of retrosynthetically interesting chemical substructure (BRICS) fragmentations [94,95].

For the ECFP approach, molecular fingerprints, i.e., fragments with a radius up to 5 bonds, are generated from each in silico ligand. These fragments were filtered to ensure a minimum size of 3 atoms, and duplicates were removed via canonical SMILES [71]. To determine their significance, fragment frequencies within basic and elevated stabilizer candidates, and regular organic ligands were compared via the following scoring function.

$$\text{Score}=\frac{f_{g}-f_{\bar{g}}}{\text{exp}\left(f_{\bar{g}}\right)}$$ (4)

where $f_{g}$ and $f_{\bar{g}}$ represent the frequency of a fragment within the stabilizer candidates (molecular glues, *g*) and the regular organic ligands (not molecular glues, $\bar{g}$).

Instead of adjacency-based fragmentation, BRICS divides molecules along bonds based on rules motivated by synthetic chemistry [95]. Inversion of this bond-breaking rule set ensures that obtained substructures can recombine to synthesize novel compounds. We used RDKit [84] for ligand fragmentation and recombination of fragments associated with RPI stabilization into novel compounds. The generated compounds and substructures from both methods were compiled into databases for filtering and de novo design (for access, see Data Availability).

#### Comparison to Chai-1 predictions

The devised workflow offers a multistep, high-throughput method that includes the in silico placement of organic ligands at RNA–protein complexes. Recently, structure prediction models became available, enabling the prediction of such ternary complexes in a one-step process. Chai-1 [54] is such a deep learning model that can generate 3-dimensional structures of macromolecular complexes with organic, drug-like compounds.

Chai-1 was queried 5 times to assemble each of the 1,313 receptor–ligand complexes—1,300 for each docked compound and an additional 13 for the experimental ligands. The input comprised the protein and RNA chains in FASTA format, and the ligands were given in SMILES format. Chai-1 was evaluated based on its ability to correctly place ligands at experimentally known binding sites and achieve congruence with workflow-predicted ligands. Therefore, we devised several metrics to quantify Chai-1’s matching quality. Since we are interested in how well Chai-1 predictions match those from our workflow, we used the best-matching assembly from 5 attempts for comparison. Confidence metrics of Chai-1 were not considered.

The $\Delta L_{\text{macro}}$ metric is used to compare Chai-1, in silico, and experimental ligands with each other, resulting in 3 metrics for each associated protein–RNA complex. This measure is calculated as the Euclidean norm between the ligands’ center of mass (COM) vectors L, with the mobile ligand being transformed according to the RMSD alignment of the macromolecule backbones, $R_{\text{macro}}$ and ${\vec{T}}_{\text{macro}}$. A COM distance measure is used, since the atomic composition mismatches for the respective ligands [63,64,78,79].

$$\begin{array}{c} \begin{array}{c} \Delta L_{\text{macro}}=\left\|\frac{\sum_{i}^{N}m_{i}\left(R_{\text{macro}}\cdot {\vec{L}}_{i}^{\mathrm{mob}}+{\vec{T}}_{\text{macro}}\right)}{\sum_{i}^{N}m_{i}}-\frac{\sum_{j}^{M}m_{j}{\vec{L}}_{j}^{\mathrm{ref}}}{\sum_{j}^{M}m_{j}}\right\|\\ \;=\left\|L_{\text{macro}}^{\mathrm{mob}}-L^{\mathrm{ref}}\right\| \end{array} \end{array}$$ (5)

Low $\Delta L_{\text{macro}}$ values indicate matching predictions. For internal reference, the cuboids’ half-diagonal was averaged across all pockets and measured 13.55 Å.

For all remaining Chai-1-modeled complexes, the ligand atoms match. Thus, 3 different RMSD metrics were calculated as well as $\Delta L_{\text{macro}}$. The ligand RMSDs after alignment on the pocket, ${\text{RMSD}}_{\text{Pckt}}$, after alignment on the interface, ${\text{RMSD}}_{\text{IF}}$, and the minimum over a trajectory, ${\text{RMSD}}_{\text{IF}}^{\text{traj}}$, are detailed below.

$${\text{RMSD}}_{\text{Pckt}}=\sqrt{\frac{1}{L}\sum_{i}^{L}{\left|\left(R_{\text{Pckt}}\cdot {\vec{L}}_{\text{Chai}}+{\vec{T}}_{\text{Pckt}}\right)-{\vec{L}}_{\mathrm{ref}}\right|}^{2}}$$ (6)

$${\text{RMSD}}_{\text{IF}}=\sqrt{\frac{1}{L}\sum_{i}^{L}{\left|\left(R_{\text{IF}}\cdot {\vec{L}}_{\text{Chai}}+{\vec{T}}_{\text{IF}}\right)-{\vec{L}}_{\mathrm{ref}}\right|}^{2}}$$ (7)

$${\text{RMSD}}_{\text{IF}}^{\text{traj}}={\text{min}}_{t_{i}}\sqrt{\frac{1}{L}\sum_{i}^{L}{\left|\left(R_{\text{IF}}\cdot {\vec{L}}_{\text{Chai}}+{\vec{T}}_{\text{IF}}\right)-{\vec{L}}_{\mathrm{ref}}\left(t_{i}\right)\right|}^{2}}$$ (8)

The pocket denotes all macromolecular backbone atoms within 6 Å of the reference ligand, and the interface is defined as the pocket and heavy ligand atoms. Low RMSD values indicate good agreement.

## Results and Discussion

The protein–RNA complexes extracted from the PDB website were systematically investigated for their druggability concerning molecular glues. A multifaceted postprocessing pipeline was implemented to facilitate this investigation. Firstly, the compounds are categorized by their in silico interaction energy into stabilizer classes and are also compared with available experimental RPI stabilizers. The topology and druggability of the detected binding pockets are analyzed, aiming to extract efficacious pocket characteristics. Moreover, molecular fragments of highly scored candidates are compiled to serve as filter criteria in combination with RDKit or as building blocks for de novo designing drug-like compounds. Finally, a complementary AI-based structure prediction model, Chai-1, compares our elaborate high-throughput workflow to a one-step machine learning solution.

### In silico ligands reproduce experimental binding sites and binding affinity profiles

**Fig. 3.**
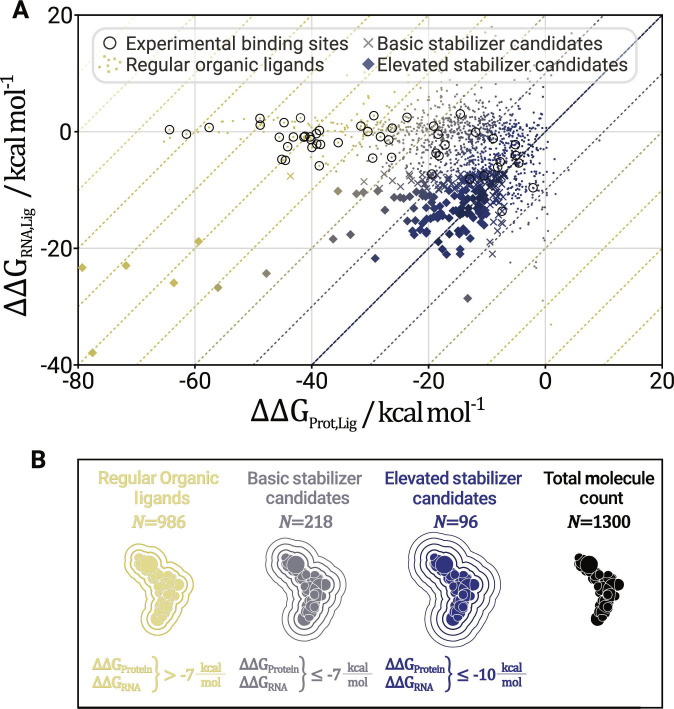
(A) Mean interaction free energy to the RNA plotted against the mean interaction free energy to the protein for 1,300 top 5 ligands. Three markers distinguish 3 compound categories: Organic ligands are marked with dots, basic stabilizer candidates with crosses, and elevated stabilizer candidates with diamonds. Each dot represents the average of a triplicate. Compounds that occupy experimental binding sites are circled (see Table 2). (B) Visualization of aforementioned ligand classes with their definitions and counts.

By applying the workflow outlined in Fig. 1, we obtained 260 interface pockets with one failure from DeepPocket due to a small and unfolded peptide structure for the PDB entry 5DEA [96]. T38DrugDB docking of the 260 interface pockets resulted in 52,000,000 docked compounds, 26,000 after docking score filtering, and 1,300 after second-rank BSA filtering. The final selection of 1,300 organic ligands was thoroughly analyzed via MD simulation and MMGB/SA. This analysis evaluates the ligands’ ∆∆G_Protein_ and ∆∆G_RNA_, enabling a more robust estimate of the stabilizing potential than plain docking. From the resulting 1,300 ligands, 218 (17%) can be considered basic stabilizers, and 96 ligands (7%) can be considered elevated stabilizer candidates (see Fig. 3). The basic stabilizer candidates were found in 57 complexes (66%), with 34 complexes (39%) featuring elevated stabilizer candidates. Thus, a large proportion of resolved complexes was found in silico to host stabilizer candidates, and the stabilizer candidates themselves represent a substantial fraction of the top-performing docked ligands. We consider this an encouraging insight into the novel field of RPI stabilizers.

In Figs. S1 to S3, a visualization of elevated stabilizer candidates with their complexes can be found. This knowledge could guide experimental drug discovery within the depicted protein–RNA interfaces.

Of the 87 investigated protein–RNA complexes, 13 contain experimentally resolved organic compounds that bind at the protein–RNA interface. The organic compounds are mostly cofactors like adenosine triphosphate (ATP), S-adenosyle methionine (SAM), or flavin mononucleotide (FMN), but also buffer components like 2-(N-morpholino)-ethanesulfonic acid (MES) and one interaction stabilizer by the name of desmethyl pateamine A (V6D) (see Table 2). These receptor–ligand complexes were utilized as experimental controls. Their spectrum of distinct biological functionalities entails the potential to demonstrate the robustness of our computational workflow. Hence, congruent, detected pockets should host in silico ligands exhibiting shapes, ESPs, and binding affinities similar to their experimental counterparts.

For 10 of the 13 PDB entries listed in Table 2, the experimental binding pocket could be reproduced by our workflow. However, 3 experimentally resolved binding sites—4GCW, 7K9D, and 2ZZM—could not be replicated (see Fig. S4). 4GCW and 7K9D had their compounds outside the pocket boundaries found by fpocket, MDpocket, and DeepPocket. The organic ligands for these 2 PDB files were identified as buffer components instead of dedicated binders. Reproducing buffer binding sites would be detrimental to the fidelity of our workflow, as these result from crystallographic methodology rather than ligand specificity. Therefore, not reproducing these experimental solutes correctly identifies their presumed binding site as true negatives. These findings contribute to the robustness of this workflow. For 2ZZM, the SAM molecule features only negligible BSA with the RNA. It would therefore be found unsuitable for RPI stabilization in the pocket evaluation step.

Concerning the remaining 10 complexes listed in Table 2, the experimental binding sites could be reproduced. Most experimental ligands for these 10 complexes represent cofactors or cosubstrates that are primarily bound to the protein molecule to support its functionality [97–104]. Exceptions to this rule are 2HVY and 6XKI. The ATP at 2HVY is a buffer component that supports crystallization by compound-mediated interactions between protein and RNA. The experimental ligand to 6XKI is the aforementioned interaction stabilizer V6D [43,105].

**Fig. 4.**
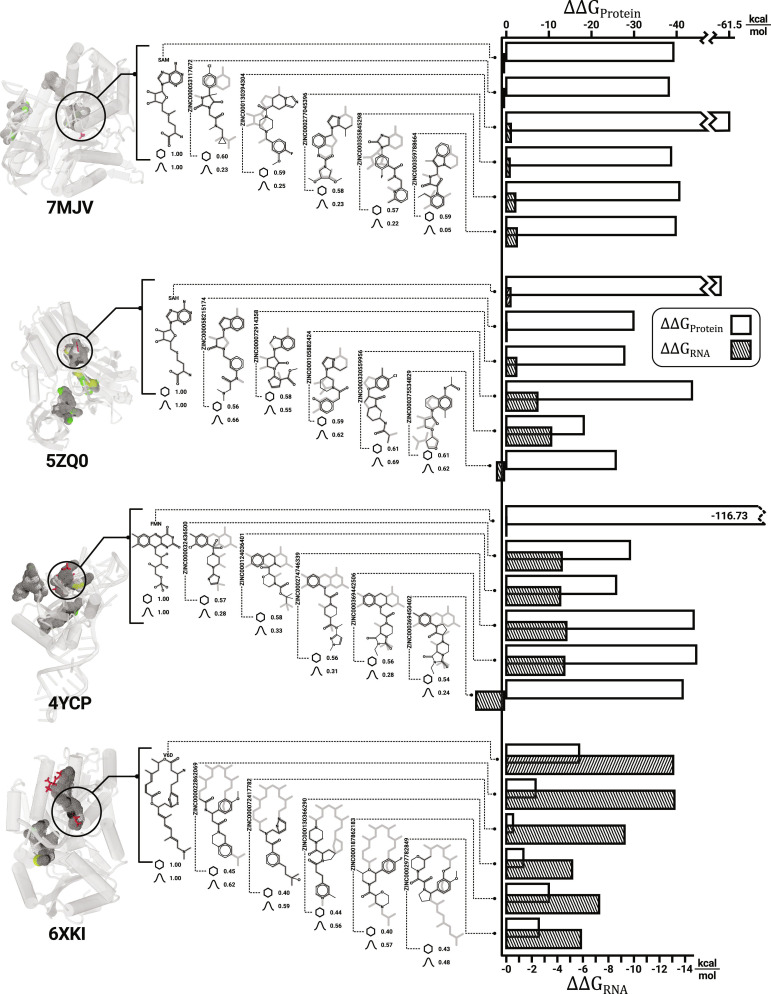
Overview of complexes with experimental binding sites. Renders of the 4 complexes 7MJV, 5ZQ0, 4YCP, and 6XKI are shown on the very left. The in silico ligands are rendered as wireframes, and the experimental ligand is shown as a red licorice representation. These are listed in the middle of the figure as black skeletal formulas with the experimental ligands’ gray scaffold in the background. The ESP and shape similarity are printed below each ligand, with the hexagon and Gaussian icon marking the shape and ESP similarity score. To the very right, the MMGB/SA-derived ∆∆*G* values are shown for the protein (white) and the RNA (shaded).

As a representative sample, 7MJV, 4YCP, 5ZQ0, and 6XKI are shown in Fig. 4. The figure contains information on the predicted binding sites and the structural comparison of the in silico and experimental ligands, including shape and ESP similarity scores, and ∆∆G_RNA_ and ∆∆G_Protein_ values obtained from MMGB/SA [83]. Each of the complexes’ results shall be briefly explained.

7MJV is a methyltransferase with SAM interacting mostly with the protein. The in silico ligands show unfavorable ∆∆G_RNA_ values, while the interaction with the protein is strongly favorable, matching the experimental ligand’s affinities. The best agreement with the experiment regarding relative binding free energies is achieved by the first in silico compound, which also shows the highest shape similarity of 0.6. The ESP similarity is quite low for this complex, with a maximum of 0.25.

A similar case is presented by 5ZQ0, a methyltransferase with the resolved binder S-adenosyl-l-homocysteine (SAH). Most noticeably, the ESP similarities of those compounds are higher by a margin, ranging around 0.55 to 0.69. This shift in ESP similarity is likely due to the positive charge at SAM’s central sulfur atom, missing for SAH. The fourth ligand features the highest shape and ESP score while exhibiting the least agreeing binding free energies. Nevertheless, the experimentally found ∆∆*G*s are qualitatively reproduced by the in silico ligands but with lesser quantitative agreement.

The experimental ligand of 4YCP exhibits a disproportionally sizeable attraction to the protein, which might result from the combination of a buried, highly charged phosphate group, contacting 2 arginine residues.

Lastly, 6XKI, the only protein–RNA complex hosting an experimental interaction stabilizer, exhibits a distinct pattern. Here, the protein and RNA attractions are balanced out immediately visible by the unprecedented extent of the shaded bars (Fig. 4). The ∆∆*G* values of the experimental ligand are matched excellently by the in silico predictions mimicking the biological activity of this ternary complex. The experimental ligand V6D has a molecular weight of approximately 541 g mol^−1^ and is therefore outside of the filter applied to create T38DrugDB, which might cause the relatively low shape scores.

In summary, the experimental binding sites are detected by the pocket algorithms in 77% of the cases, with the 23% undetected pockets featuring either buffer components or insufficient interface coverage. The detected pockets’ structural environment and biological functions define the chemical constraints that any accommodated ligand must satisfy, including those related to the mean interaction free energy. Therefore, reproducing the affinity profiles of the experimental ligands with our in silico predictions validates the workflow’s ability to generate realistic ligands that could exert biological activity for a given interface pocket. The validation concerning the PDB entry 6XKI is especially relevant. This complex binds an experimental interaction stabilizer, and fittingly, 2 of its in silico ligands fall within our definition of a basic stabilizer candidate. Metrics for the remaining experimental complexes are in the Supplementary Materials (see Figs. S5 to S10).

### Interface pocket detection

**Fig. 5.**
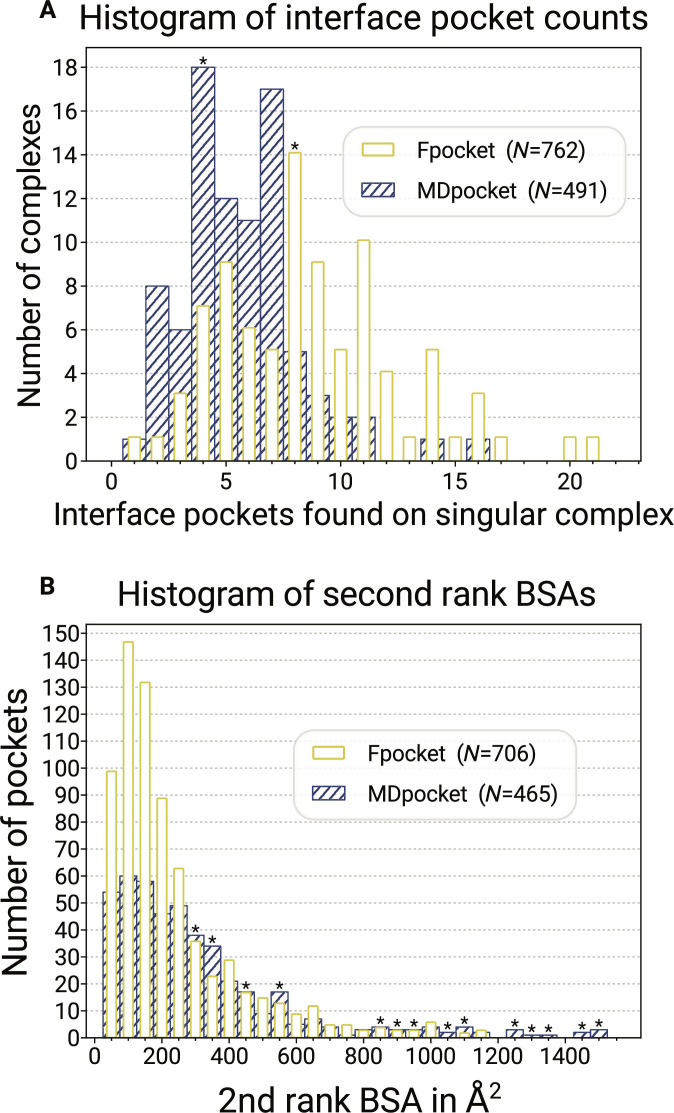
Histograms comparing the second-rank BSA and the total number of interface pockets found per complex for fpocket and MDpocket. (A) Number of interface pockets found per complex. Pockets with a second-rank BSA below 20 Å^2^ are excluded. MDpocket finds 4 interface pockets on a singular complex most frequently (true for 18 complexes), while fpocket finds 8 interface pockets most frequently (true for 14 complexes), which are indicated by asterisk symbols. (B) Pocket counts by second-rank BSA. Pockets with second-rank BSA below 50 Å^2^ are excluded. Fpocket finds 706 pockets compared to 465 pockets found by MDpocket. For fpocket, the distribution is visibly elevated around 50 to 250 Å^2^. Asterisk symbols indicate where MDpocket surpasses fpocket’s counts.

After evaluating the validity of our in silico approach, we analyzed pocket detection algorithms and assessed how detection and filtering affected a pocket’s capacity to host stabilizer candidates. The results obtained from fpocket and MDpocket are compared to assess qualitative differences in pocket detections between static crystal structures and time-series data. A direct comparison of the respective pocket statistics is visualized in Fig. 5. Concerning their general ability to detect pockets, both algorithms find interface pockets for every investigated complex. MDpocket finds up to 16 pockets for some complexes, and fpocket finds up to 22 (see Fig. 5A). The second-rank BSA of these pockets indicates how well ligands may interact with each binding partner. Therefore, all pockets’ second-rank BSA were compiled into Fig. 5B. Pockets with low second-rank BSA dominate, and many pockets feature a BSA below 100 for fpocket and MDpocket. The general trends are similar for the two, with fpocket featuring more pockets in total.

**Fig. 6.**
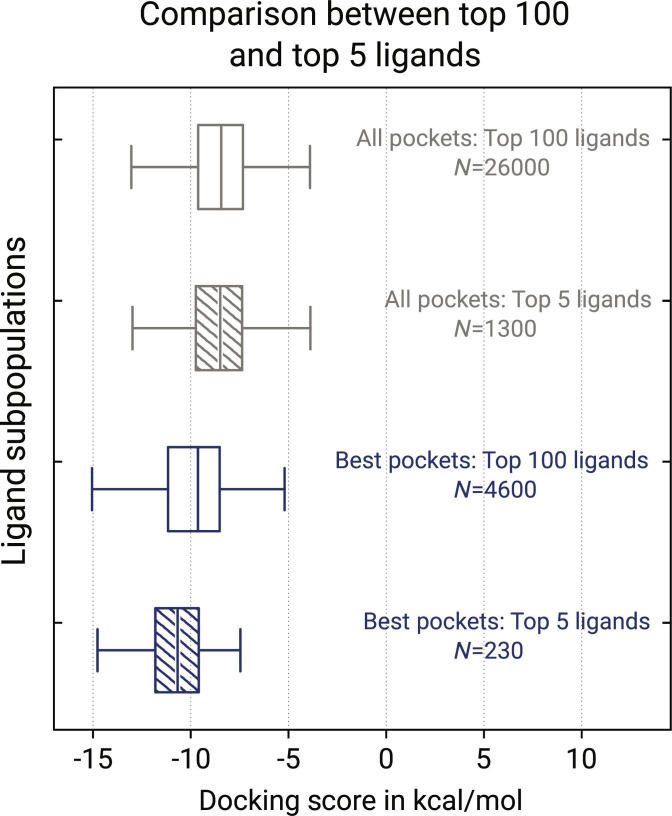
Comparison between different ligand subpopulations regarding the docking score. The ligand subpopulations are classified 2-fold: pocket-wise and by ligand ranking. Subpopulations ranked in the top 100 are plain, while the top 5 are shaded. Subpopulations from all pockets are light gray, while those containing elevated stabilizer candidates are dark blue, herein called best pockets. The subpopulations’ docking score distributions are shown as box plots with the first, second, and third quantiles, and the whiskers denote 1.5 times the interquartile range. The total number of raw data points is printed beneath the labels. Compared to all pockets, best pockets generally exhibit lower docking scores.

Next, we investigated how BSA-based top 5 filtering affects the average docking score across ligand subpopulations stratified by pocket cohort. We defined 4 subpopulations based on 2 criteria: whether pockets contained at least one elevated stabilizer candidate and the filtering level (top 100 or top 5; see Fig. 6). We observe little to no effect when all pockets are considered. However, top 5 filtering induces a considerable shift in stabilizer-containing pockets toward lower docking scores. This shift hints at a positive correlation between docking scores, second-rank BSA, and a pocket’s capacity to host stabilizer candidates.

**Fig. 7.**
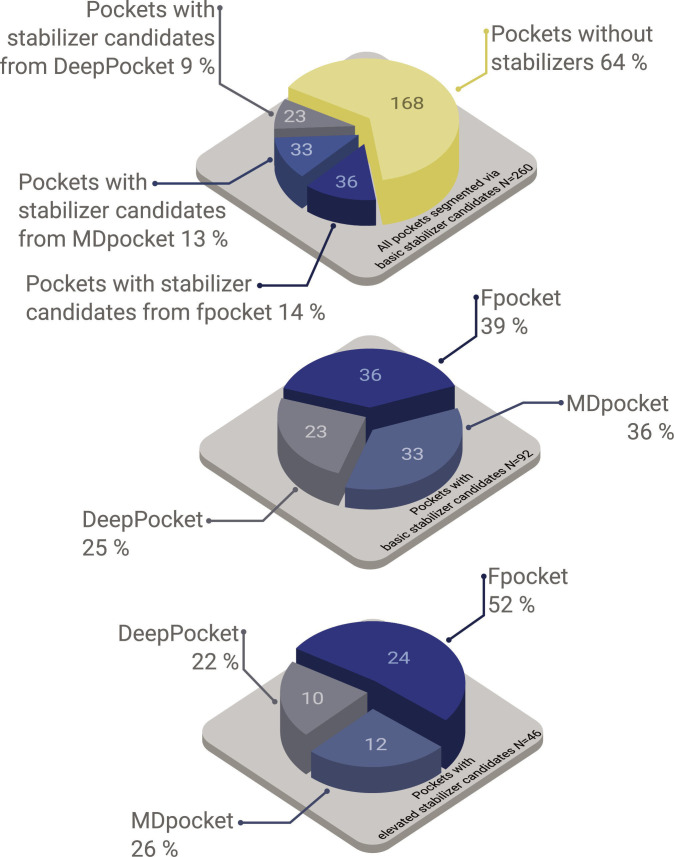
Statistics for the performance of interface pockets for each pocket algorithm regarding the presence of stabilizer candidates.

Fpocket generated 36 pockets that hosted one or more basic stabilizer candidates and 24 pockets with elevated stabilizer candidates. MDpocket generated 33 and 12 such pockets, while DeepPocket generated 23 and 10. Notably, the order in which the pocket algorithms were applied is the same rank order. Fpocket detects the pockets first, then MDpocket and DeepPocket last. If the latter detected the same pocket as the former, the next most promising pocket would be selected by it instead; therefore, a clear ranking of the pocket algorithms cannot be drawn (see Fig. 7).

### Random forest and feature importance: Docking score and BSA are most indicative

Various metrics can be used to characterize a binding site, but figuring out which are indicative of pocket druggability remains challenging. One goal of the presented study was to constitute an interface pocket’s druggability from a priori molecular descriptors. Thus, 77 pocket features were compiled for training a random forest classifier to predict pocket druggability, herein defined as a pocket’s propensity to accommodate a stabilizer candidate. Two models were trained on the basic and elevated stabilizer candidates, respectively. Based on their ROC-AUC and sensitivity, the random forest classifiers can be considered informative predictors, which facilitates a sound feature importance analysis.

**Fig. 8.**
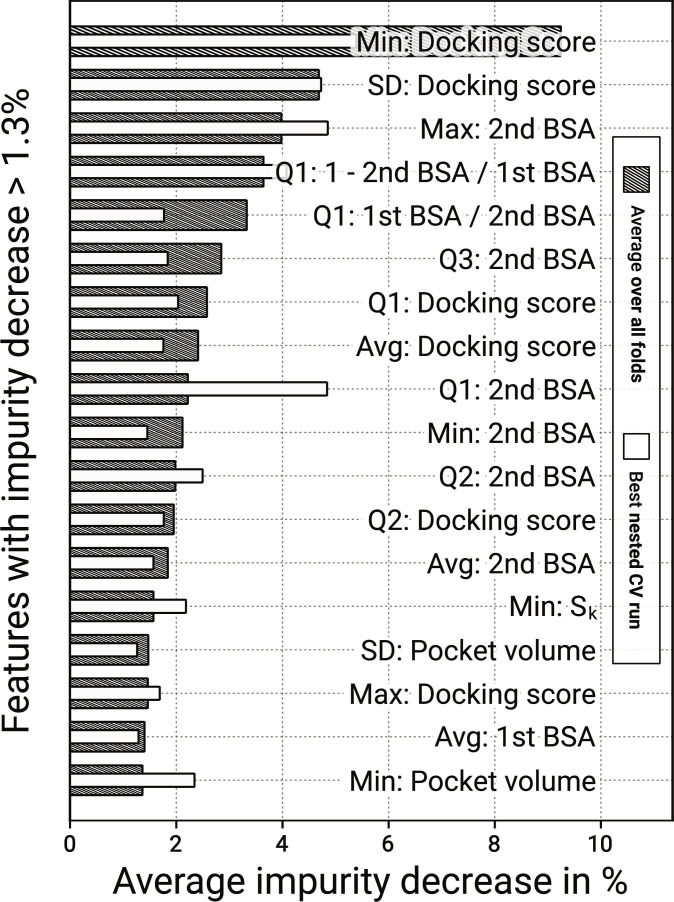
Feature importance results from the random forest classifier trained on the basic stabilizer candidates. The shaded bars depict the average results across preprocessing grid iterations, while the white bars show the nested CV loop that achieved the highest balanced accuracy. The minimum docking score—i.e., the most favorable docking score—is the most predictive feature.

The feature importance reveals that the docking scores’ minimum and standard deviation are the most important features by a margin (see Fig. 8). The impurity decreases are 9.3% and 4.7%, with 1.3% being the average impurity decrease per feature. The next-highest feature concerns the maximum second-rank BSA achieved by any docked compound, which is 4.0%. The only impactful features derived by MD simulations—instead of docking statistics—are the minimum translational order parameter, $S_{k}$, the pocket volume’s standard deviation, and minimum, which rank 14th, 15th, and 18th, respectively, with impurity decreases at 1.6%, 1.5%, and 1.4%. The results from the elevated stabilizer candidates confirm these findings (see Figs. S11 and S12).

These features’ low rankings indicate that evaluating pocket hydration and fluctuation properties with MD is not impactful for the delineated workflow. Instead, a sensible approach to evaluating pocket druggability is to dock random drug-like compounds and then keep the pockets with sufficiently favorable, small docking scores and large second-rank BSAs. This study does not address the likelihood of in silico ligands accessing or diffusing into a pocket, a process expected to depend more strongly on hydration dynamics and conformational fluctuations observable in apo-complex simulations.

### Chemical fragmentation analysis: Molecular glues feature distinct set of chemical fragments

With an understanding of pocket features that aid druggability, the question remains: Which compound features facilitate RPI stabilization? We decomposed the identified stabilizer candidates into chemical substructures via ECFP and BRICS fragmentation to tackle this issue. The created fragment libraries are made publicly available (see Data Availability). The ECFP fragments are basic SMILES strings that can filter an existing database of organic compounds. In contrast, the BRICS database can be used to generate novel compounds by randomly combining BRICS fragments.

**Fig. 9.**
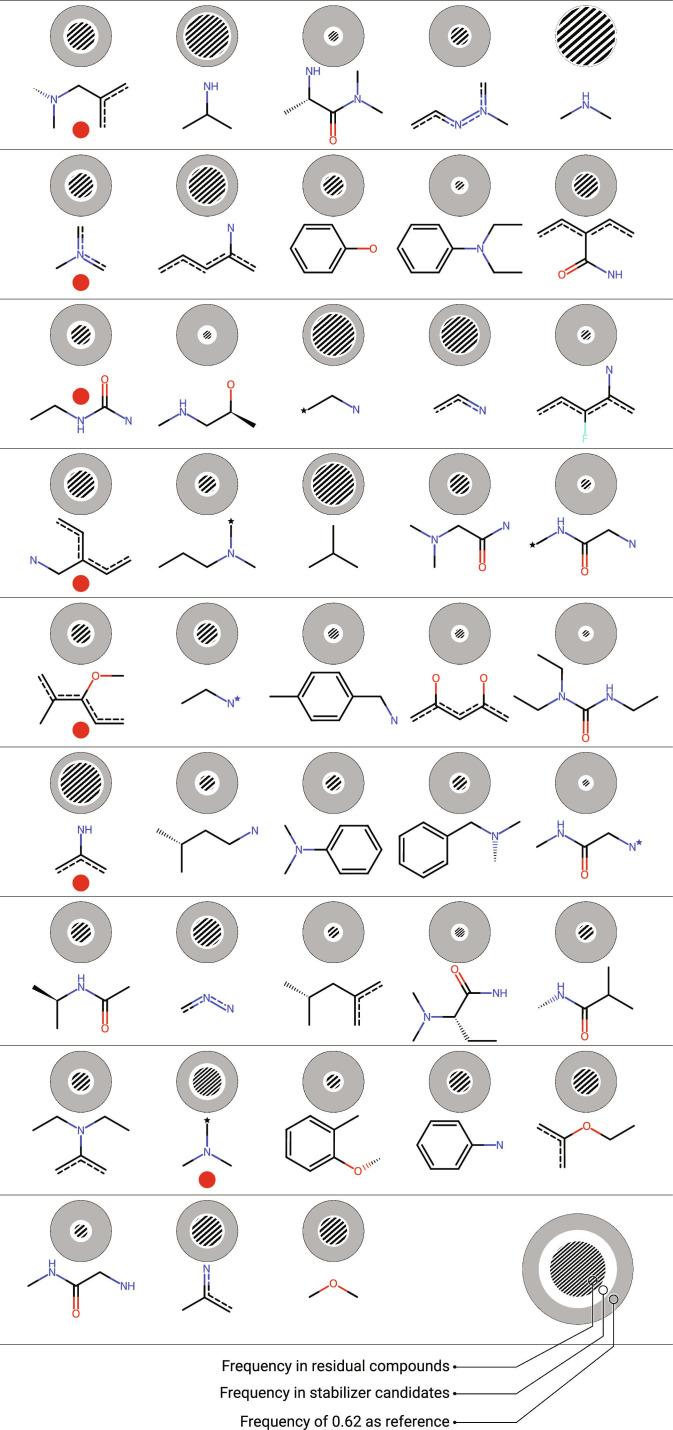
Highly scored compound fragments derived from the elevated stabilizer candidates. The frequencies of ECFP fragments within the drug candidates are shown as the white circular area, while the frequencies within the residual compounds are shaded circles. The gray circle with constant circumference corresponds to the maximum observed fragment frequency of 0.62 and serves as a reference. The red dots mark compounds with high discriminatory potential. Stars indicate terminal aromatic atoms.

The in silico ligands evaluated via MMGB/SA were split into (elevated) stabilizer candidates and residual compounds, in order to arrive at a database of SMILES strings that can be used as a compound filter for large-scale screening. Their fragments were generated and scored according to Eq. (4). The scoring ensures that the fragments discriminate well between elevated stabilizer candidates and the residual compounds. The 43 highest-scoring fragments suffice to map the elevated stabilizer candidates, while 881 would be required to map the remainder. These 43 fragments are shown in Fig. 9. Furthermore, the 7 most discriminating fragments manage to represent 80% of the elevated stabilizer candidates while only mapping 57% of the other organic compounds (compare Fig. 9; fragments marked with a red dot). It can, therefore, be assumed that the presented ECFP fragments have discriminatory potential regarding database filtering. Since these fragments resulted exclusively from statistical analysis, there is limited mechanistic insight within these findings.

On the other hand, the BRICS-derived fragments do not require scoring as conducted for the ECFP fragments. The fragments shown in Fig. S13 comprise the complete set that could be generated from the group of elevated stabilizer candidates and amounts to 213 fragments. The use case of the BRICS substructures is to recombine them into novel molecules that possibly share the RPI stabilizing properties found in the elevated stabilizer candidates. The recombination of BRICS fragments obeys rules derived from organic synthesis and is therefore experimentally and computationally viable. We created a 4-million-compound database via computational recombination using RDKit’s BRICS module [84]. This database contains all unique recombinations involving 3 BRICS substructures. It is made openly available alongside the 213 fragments and instructions for using this information for de novo drug design (see Data Availability).

### Chai-1 excels on experimental data but conflicts with in silico predictions

The results obtained from our workflow were compared with the one-step structure prediction model Chai-1 [54]. The comparison to experimental data utilizes the COM distance $\Delta L_{\text{macro}}$ to compare the Chai-1, in silico, and experimental ligand placements among each other [see Eq. (5)]. The protein–RNA complexes with in silico ligands are compared to Chai-1 predictions via $\Delta L_{\text{macro}}$ and the 3 RMSD metrics [see Eq. (6)].

**Fig. 10.**
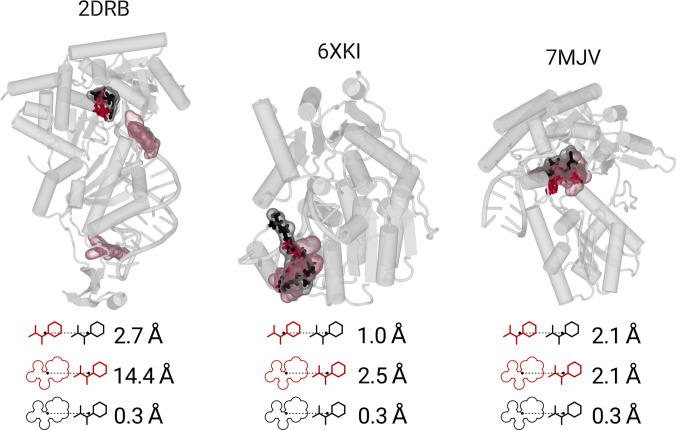
Visualization of the complexes 2DRB, 6XKI, and 7MJV. The images contain the experimental ligand (black licorice), the in silico ligands (red licorice), and the Chai-1 placements (transparent surface in corresponding color). Below each render, the $\Delta L_{\text{macro}}$ averages are shown, with the icons resembling each representation. The macromolecules are transparent gray.

The results of the experimental comparison are shown in Fig. 10 for 2DRB, 6XKI, and 7MJV. The Chai-1 model produces $\Delta L_{\text{macro}}$ values of 0.3 Å for all experimental ligands. However, this precision likely reflects Chai-1’s exposure to PDB structures during training and should not be interpreted as generalizable predictive accuracy. A similar finding is reported in a recent preprint by Škrinjar et al. [106], which shows that high-quality predictions are largely confined to complexes with close similarity to the training set, while novel molecules or binding pockets remain challenging for Chai-1 and related structure prediction models. The workflow-derived in silico ligands are placed near the experimental binders, due to apposite pocket detection with average $\Delta L_{\text{macro}}$ values of 2.7, 1.0, and 2.1 Å. Greater variance is observed for the $\Delta L_{\text{macro}}$ between in silico and Chai-1 ligand placements. Here, results are 14.4, 2.5, and 2.1 Å, a pattern consistent with the remaining complexes (see Fig. S14). To summarize, we find highly accurate Chai-1 placements of experimental ligands and varying overlap with the in silico placements. Like our workflow, Chai-1 fails to match the experimental ligands of 4GCW and 7K9D, which represent buffer components.

**Fig. 11.**
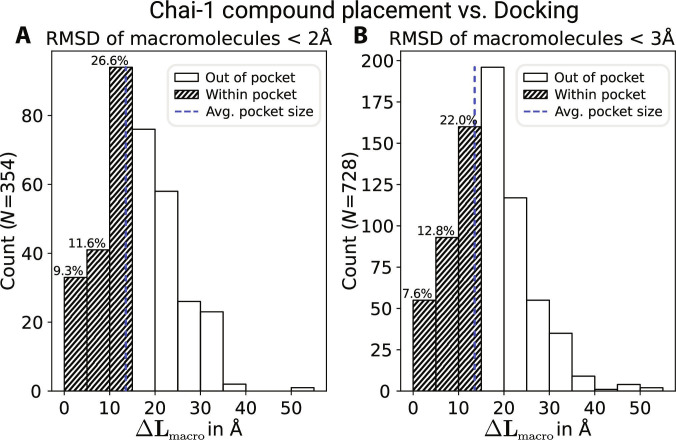
Histogram of the COM distances, $\Delta L_{\text{macro}}$, measured between the Chai-1 and our workflow’s in silico ligand placements. The dashed blue line indicates the average pocket half-diagonal. Bins to the left of this threshold are labeled with their percentages. (A) Only Chai-1 placements with an alignment RMSD below 2 Å. (B) Only Chai-1 placements with an alignment RMSD below 3 Å. *Y*-axis labels indicate subset sizes.

The non-experimental comparison was conducted between the workflow placements of the in silico ligands and their corresponding Chai-1 placements. Concerning the $\Delta L_{\text{macro}}$, the alignment RMSD was required to be lower than 2 or 3 Å since the congruence of macromolecule backbone is imperative for the validity of subsequent ligand placements. Hence, ligand placements for alignment scores above 3 Å were omitted from analysis. Those results are compiled into 2 histograms in Fig. 11. Agreeing ligand placement can be assumed for $\Delta L_{\text{macro}}$ values smaller than 5 Å, which is the case for 9.3% and 7.6%. Compared against the average pocket half-diagonal of 13.55 Å, 59.6% or 64.7% of placements lie above it and can thus be safely considered to be in disagreement. These findings indicate that accurate macromolecule prediction correlates with Chai-1’s ligand placement.

For further analysis, 3 RMSD measurements were calculated: ${\text{RMSD}}_{\text{pocket}}$, ${\text{RMSD}}_{\text{IF}}$, and ${\text{RMSD}}_{\text{IF}}^{\text{traj}}$. The alignment quality threshold for each of the RMSD measurements was selected to be 5 Å. Ligand placement of accepted structures is considered good if the respective RMSD is below 5 Å and in disagreement if it is above 10 Å (see Table 3).

**Table 3.** The RMSD comparison between Chai-1 predictions and our workflow. In all 3 cases, an alignment quality threshold of 5 Å is chosen, resulting in subsets ($N_{\mathrm{Acc}}$) of accepted Chai-1 predictions, which are categorized in good RMSD fits ($N_{<5Å}$) and tolerable RMSD fits ($N_{<10Å}$). Further, the percentages ($p_{<Å}$) are given within all 1,300 protein–RNA–ligand complexes.

Property	$N_{\mathrm{Acc}}$	$N_{<10Å}$	$N_{<5Å}$	$p_{<10Å}$	$p_{<5Å}$
${\text{RMSD}}_{\text{pocket}}$	977	152	14	11.7%	1.1%
${\text{RMSD}}_{\text{IF}}$	172	162	19	12.5%	1.5%
${\text{RMSD}}_{\text{IF}}^{\text{traj}}$	222	214	63	16.5%	4.8%

The ${\text{RMSD}}_{\text{pocket}}$ yields the most accepted complexes (977). However, it also shows the fewest accepted ligand placements. Thus, Chai-1 predicts most ligands outside the pocket. The ${\text{RMSD}}_{\text{IF}}$ results in a far lower number of accepted ligand–receptor complexes (172). Taking all trajectory frames into account with ${\text{RMSD}}_{\text{IF}}^{\text{traj}}$ results in a higher number of accepted structures (222) and the highest number of well-placed organic ligands (63). However, compared to the total number of available protein–RNA–ligand complexes, the proportion of agreeing Chai-1 predictions is quite low. The highest percentages are also produced by the ${\text{RMSD}}_{\text{IF}}^{\text{traj}}$ with 4.8% and 16.5%.

This analysis reveals discrepancies between Chai-1’s one-step approach and our workflow predictions. The $\Delta L_{\text{macro}}$ comparison scores 33 or 55 ligand–receptor complexes within 5 Å of our in silico predictions (Fig. 11). The RMSD-based analysis of static data yielded 14 and 19 correctly positioned ligands, indicating that accurate pose prediction is more limited for Chai-1 than binding site prediction (Table 3). This finding aligns with our expectations, as identifying the correct binding site is a prerequisite for accurate pose determination. This number increases to 63 for dynamic, trajectory-wide structure comparisons, suggesting that the sampled ligand configurations occasionally overlap more closely with the Chai-1 predictions. Thus, substantially more Chai-1 ligand predictions are physically feasible than suggested by the static comparisons. Nevertheless, the high failure rate observed across all comparative geometric measurements thus far discourages the use of Chai-1 as an augmentation for our virtual screening workflow.

## Conclusion

The interfaces of protein–RNA complexes often contain cavities that can potentially bind interaction-stabilizing molecules. Complex-wise, extensive docking of putative ligands indicates basic interaction stabilizer candidates for 66% and elevated candidates for 39% of complexes according to a favorable MMGB/SA interaction with the RNA and the protein. Pocket-wise, across a set of 260 protein–RNA interface pockets, 35% to 18% were identified as suitable for accommodating basic or elevated stabilizer candidates. Some docked ligands with favorable scores showed notable similarity to experimental ligands, underscoring the usefulness of the employed computational workflow.

The analysis of pocket detection algorithms and the pocket feature importance analysis revealed a novel perspective on assessing pocket druggability. We found that druggability can also be evaluated by docking a large number of drug-like compounds (such as those deposited in T38DrugDB) into each pocket. The pockets can then be ranked by the most favorable docking scores and the docking score variances.

The chemical space analysis revealed key fragments overrepresented in stabilizer candidates, which enables the refinement of compound libraries for targeted screening and de novo drug design.

The Chai-1 predictions show that several stabilizer candidates yield bound configurations very close to the docking predictions. Also, Chai-1 confirmed the exclusively affirmative results obtained for the 6XKI complex, which contains this study’s only experimentally resolved RPI stabilizer. However, the discrepancy in most ligand placement comparisons is greater than 10 Å, effectively indicating mismatches with Chai-1. 

Although challenges remain in the AI-guided one-step reproduction of in silico binders and in elucidating the structure–activity relationship of expounded stabilizer candidates, these results highlight the potential of the devised methodology.

Protein–RNA interactions are essential for many biological processes such as transcription, posttranscriptional modification, translation, and genetic regulation, and also play a role in various diseases [107–112]. Our study indicates that a significant number of involved protein–RNA interfaces are amenable for stabilizer design. Thus, further exploration of RPI stabilization is encouraged to better understand pocket detection and druggability with small organic ligands.

Several interesting directions for future studies could add insight and momentum to the field of RPI stabilizers including binding experiments on the proposed in silico stabilizer candidates.

Another direction could be a large-scale comparison of generalized transformer models, such as AlphaFold 3, Chai-1, and Boltz-2. As in this study, ligand placement quality and the optimization of model presets should be the focus of such an endeavor. Performant predictive modeling could provide a validation or refinement layer between high-throughput virtual screening and MD-powered MMGB/SA calculations. The random forest classifier trained to predict pocket druggability is another valuable approach that warrants further exploration by applying and testing alternative machine learning architectures. These in particular benefit from expanding the dataset of protein–RNA complexes, preferably with bound drug-like ligands.

## Data Availability

The datasets that resulted from the research outlined in this publication are made available in several different repositories. The T38DrugDB containing 34,000,000 medicinally relevant compounds can be accessed via github.com/Foly93/T38DrugDB. The databases containing the molecular fragments of the ECFP and BRICS analysis for filtering and de novo drug discovery are deposited under github.com/Foly93/RPIS_FragmentationLibraries. Further research data, parameters, scripts, and other software prepared to generate the presented results are available in github and can be requested by messaging https://github.com/Foly93/. The ligand–receptor complexes containing elevated stabilizer candidates are showcased in Fig. S1.

## Supplementary Materials

Supplementary 1 Notes S1 to S4 Figs. S1 to S14 Table S1

## References

[1] Lu H, Zhou Q, He J, Jiang Z, Peng C, Tong R, Shi J. Recent advances in the development of protein-protein interactions modulators: Mechanisms and clinical trials. Signal Transduct Target Ther. 2020;5(1):1–23. 32968059 10.1038/s41392-020-00315-3 PMC7511340

[2] Camps-Fajol C, Cavero D, Minguillón J, Surrallés J. Targeting protein-protein interactions in drug discovery: Modulators approved or in clinical trials for cancer treatment. Pharmacol Res. 2025;211: Article 107544. 39667542 10.1016/j.phrs.2024.107544

[3] Modell AE, Blosser SL, Arora PS. Systematic targeting of protein-protein interactions. Trends Pharmacol Sci. 2016;37(8):702–713. 27267699 10.1016/j.tips.2016.05.008 PMC4961577

[4] Mabonga L, Kappo AP. Protein-protein interaction modulators: Advances, successes and remaining challenges. Biophys Rev. 2019;11(4):559–581. 31301019 10.1007/s12551-019-00570-x PMC6682198

[5] Petta I, Lievens S, Libert C, Tavernier J, De Bosscher K. Modulation of protein-protein interactions for the development of novel therapeutics. Mol Ther. 2016;24(4):707–718. 26675501 10.1038/mt.2015.214 PMC4886928

[6] Andrei SA, Sijbesma E, Hann M, Davis J, O’Mahony G, Perry MWD, Karawajczyk A, Eickhoff J, Brunsveld L, Doveston RG, et al. Stabilization of protein-protein interactions in drug discovery. Expert Opin Drug Discov. 2017;12(9):925–940. 28695752 10.1080/17460441.2017.1346608

[7] Zarzycka B, Kuenemann MA, Miteva MA, Nicolaes GAF, Vriend G, Sperandio O. Stabilization of protein-protein interaction complexes through small molecules. Drug Discov Today. 2016;21(1):48–57. 26434617 10.1016/j.drudis.2015.09.011

[8] Dewey JA, Delalande C, Azizi S-A, Lu V, Antonopoulos D, Babnigg G. Molecular glue discovery: Current and future approaches. J Med Chem. 2023;66(14):9278–9296. 37437222 10.1021/acs.jmedchem.3c00449 PMC10805529

[9] Rui H, Ashton KS, Min J, Wang C, Potts PR. Protein-protein interfaces in molecular glue-induced ternary complexes: Classification, characterization, and prediction. RSC Chem Biol. 2023;4(3):192–215. 36908699 10.1039/d2cb00207h PMC9994104

[10] Borel JF, Feurer C, Gubler HU, Stähelin H. Biological effects of cyclosporin A: A new antilymphocytic agent. Agents Actions. 1994;43(3):179–186. 7725970 10.1007/BF01986686

[11] Laupacis A, Keown PA, Ulan RA, McKenzie N, Stiller CR. Cyclosporin A: A powerful immunosuppressant. Can Med Assoc J. 1982;126(9):1041–1046. 7074504 PMC1863293

[12] Bierer BE, Holländer G, Fruman D, Burakoff SJ. Cyclosporin A and FK506: Molecular mechanisms of immunosuppression and probes for transplantation biology. Curr Opin Immunol. 1993;5(5):763–773. 7694595 10.1016/0952-7915(93)90135-f

[13] Juvvadi PR, Fox D, Bobay BG, Hoy MJ, Gobeil SMC, Venters RA, Chang Z, Lin JJ, Averette AF, Cole DC, et al. Harnessing calcineurin-FK506-FKBP12 crystal structures from invasive fungal pathogens to develop antifungal agents. Nat Commun. 2019;10(1):4275. 31537789 10.1038/s41467-019-12199-1 PMC6753081

[14] Cardenas ME, Zhu D, Heitman J. Molecular mechanisms of immunosuppression by cyclosporine, FK506, and rapamycin. Curr Opin Nephrol Hypertens. 1995;4(6):472. 8591053 10.1097/00041552-199511000-00002

[15] Schreiber SL, Crabtree GR. The mechanism of action of cyclosporin A and FK506. Immunol Today. 1992;13(4):136–142. 1374612 10.1016/0167-5699(92)90111-J

[16] Rowinsky EK, Donehower RC. Paclitaxel (Taxol). N Engl J Med. 1995;332(15):1004–1014. 7885406 10.1056/NEJM199504133321507

[17] Bernabeu E, Cagel M, Lagomarsino E, Moretton M, Chiappetta DA. Paclitaxel: What has been done and the challenges remain ahead. Int J Pharm. 2017;526(1):474–495. 28501439 10.1016/j.ijpharm.2017.05.016

[18] Abu Samaan TM, Samec M, Liskova A, Kubatka P, Büsselberg D. Paclitaxel’s mechanistic and clinical effects on breast cancer. Biomolecules. 2019;9(12):789. 31783552 10.3390/biom9120789 PMC6995578

[19] Sakamoto KM, Kim KB, Kumagai A, Mercurio F, Crews CM, Deshaies RJ. PROTACs: Chimeric molecules that target proteins to the Skp1-Cullin-F box complex for ubiquitination and degradation. Proc Natl Acad Sci USA. 2001;98(15):8554–8559. 11438690 10.1073/pnas.141230798 PMC37474

[20] Yu X, Li D, Kottur J, Shen Y, Kim HS, Park K-S, Tsai Y-H, Gong W, Wang J, Suzuki K, et al. A selective WDR5 degrader inhibits acute myeloid leukemia in patient-derived mouse models. Sci Transl Med. 2021;13(613):eabj1578. 34586829 10.1126/scitranslmed.abj1578 PMC8500670

[21] Corson TW, Aberle N, Crews CM. Design and applications of bifunctional small molecules: Why two heads are better than one. ACS Chem Biol. 2008;3(11):677–692. 19112665 10.1021/cb8001792 PMC2925120

[22] Chamberlain PP, Hamann LG. Development of targeted protein degradation therapeutics. Nat Chem Biol. 2019;15(10):937–944. 31527835 10.1038/s41589-019-0362-y

[23] Deshaies RJ. Prime time for PROTACs. Nat Chem Biol. 2015;11(9):634–635. 26284668 10.1038/nchembio.1887

[24] Békés M, Langley DR, Crews CM. PROTAC targeted protein degraders: The past is prologue. Nat Rev Drug Discov. 2022;21(3):181–200. 35042991 10.1038/s41573-021-00371-6 PMC8765495

[25] Pettersson M, Crews CM. PROteolysis TArgeting Chimeras (PROTACs)—Past, present and future. Drug Discov Today Technol. 2019;31:15–27. 31200855 10.1016/j.ddtec.2019.01.002 PMC6578591

[26] Ma N, Bhattacharya S, Muk S, Jandova Z, Schmalhorst PS, Ghosh S, Le K, Diers E, Trainor N, Farnaby W, et al. Frustration in the protein-protein interface plays a central role in the cooperativity of protac ternary complexes. Nat Commun. 2025;16(1):8595. 41022846 10.1038/s41467-025-63713-7 PMC12480974

[27] Konstantinidou M, Arkin MR. Molecular glues for protein-protein interactions: Progressing toward a new dream. Cell Chem Biol. 2024;31(6):1064–1088. 38701786 10.1016/j.chembiol.2024.04.002 PMC11193649

[28] Chen S-Y, Solazzo R, Fouché M, Roth H-J, Dittrich B, Riniker S. Cooperative free energy: Induced protein-protein interactions and cooperative solvation in ternary complexes. J Chem Theory Comput. 2025;9(21):8557–8570. 10.1021/acs.jctc.5c00736 PMC12424184 40832906

[29] Wu H, Yao H, He C, Jia Y, Zhu Z, Xu S, Li D, Xu J. Molecular glues modulate protein functions by inducing protein aggregation: A promising therapeutic strategy of small molecules for disease treatment. Acta Pharm Sin B. 2022;12(9):3548–3566. 36176907 10.1016/j.apsb.2022.03.019 PMC9513498

[30] Holdgate GA, Bardelle C, Berry SK, Lanne A, Cuomo ME. Screening for molecular glues—Challenges and opportunities. SLAS Discovery. 2024;29(2): Article 100136. 38104659 10.1016/j.slasd.2023.12.008

[31] Roos M, Pradère U, Ngondo RP, Behera A, Allegrini S, Civenni G, Zagalak JA, Marchand J-R, Menzi M, Towbin H, et al. A small-molecule inhibitor of LIN28. ACS Chem Biol. 2016;11(10):2773–2781. 27548809 10.1021/acschembio.6b00232

[32] Tsialikas J, Romer-Seibert J. LIN28: Roles and regulation in development and beyond. Development. 2015;142(14):2397–2404. 26199409 10.1242/dev.117580

[33] Brennan CM, Steitz JA. HuR and mRNA stability. Cell Mol Life Sci. 2001;58(2):266–277. 11289308 10.1007/PL00000854 PMC11146503

[34] Meisner N-C, Hintersteiner M, Mueller K, Bauer R, Seifert J-M, Naegeli H-U, Ottl J, Oberer L, Guenat C, Moss S, et al. Identification and mechanistic characterization of low-molecular-weight inhibitors for HuR. Nat Chem Biol. 2007;3(8):508–515. 17632515 10.1038/nchembio.2007.14

[35] Schultz CW, Preet R, Dhir T, Dixon DA, Brody JR. Understanding and targeting the disease-related RNA binding protein human antigen R (HuR). WIREs RNA. 2020;11(3): Article e1581. 31970930 10.1002/wrna.1581 PMC7482136

[36] Tailor D, Resendez A, Garcia-Marques FJ, Pandrala M, Going CC, Bermudez A, Kumar V, Rafat M, Nambiar DK, Honkala A, et al. Y box binding protein 1 inhibition as a targeted therapy for ovarian cancer. Cell Chem Biol. 2021;28(8):1206–1220.e6. 33713600 10.1016/j.chembiol.2021.02.014 PMC12950255

[37] Byun WG, Lim D, Park SB. Small-molecule modulators of protein-RNA interactions. Curr Opin Chem Biol. 2022;68: Article 102149. 35533626 10.1016/j.cbpa.2022.102149

[38] Hermann T. Strategies for the design of drugs targeting RNA and RNA-protein complexes. Angew Chem Int Ed Engl. 2000;39(11):1890–1904. 10940979 10.1002/1521-3773(20000602)39:11<1890::aid-anie1890>3.0.co;2-d

[39] Childs-Disney JL, Yang X, Gibaut QMR, Tong Y, Batey RT, Disney MD. Targeting RNA structures with small molecules. Nat Rev Drug Discov. 2022;21(10):736–762. 35941229 10.1038/s41573-022-00521-4 PMC9360655

[40] Li A, Bouhss A, Clément M-J, Bauvais C, Taylor JP, Bollot G, Pastré D. Using the structural diversity of RNA: Protein interfaces to selectively target RNA with small molecules in cells: Methods and perspectives. Front Mol Biosci. 2023;10: Article 1298441. 38033386 10.3389/fmolb.2023.1298441 PMC10687564

[41] Janprasert J, Satasook C, Sukumalanand P, Champagne DE, Isman MB, Wiriyachitra P, Towers GHN. Rocaglamide, a natural benzofuran insecticide from Aglaia odorata. Phytochemistry. 1992;32(1):67–69.

[42] Dang Y, Kedersha N, Low W-K, Romo D, Gorospe M, Kaufman R, Anderson P, Liu JO. Eukaryotic initiation factor 2-independent pathway of stress granule induction by the natural product pateamine A. J Biol Chem. 2006;281(43):32870–32878. 16951406 10.1074/jbc.M606149200

[43] Naineni SK, Liang J, Hull K, Cencic R, Zhu M, Northcote P, Teesdale-Spittle P, Romo D, Nagar B, Pelletier J. Functional mimicry revealed by the crystal structure of an eIF4A:RNA complex bound to the interfacial inhibitor, desmethyl pateamine A. Cell Chem Biol. 2021;28(6):825–834.e6. 33412110 10.1016/j.chembiol.2020.12.006 PMC8626061

[44] Ghidini A, Cléry A, Halloy F, Allain FHT, Hall J. RNA-PROTACs: Degraders of RNA-binding proteins. Angew Chem Int Ed Engl. 2021;133(6):3200–3206. 10.1002/anie.202012330 PMC7898822 33108679

[45] Dohno C, Kimura M, Fujiwara Y, Nakatani K. Photoswitchable molecular glue for RNA: Reversible photocontrol of structure and function of the ribozyme. Nucleic Acids Res. 2023;51(18):9533–9541. 37615580 10.1093/nar/gkad690 PMC10570050

[46] Douglass EFJ, Miller CJ, Sparer G, Shapiro H, Spiegel DA. A comprehensive mathematical model for three-body binding equilibria. J Am Chem Soc. 2013;135(16):6092–6099. 23544844 10.1021/ja311795d PMC3717292

[47] Tang R, Chen P, Wang Z, Wang L, Hao H, Hou T, Sun H. Characterizing the stabilization effects of stabilizers in protein-protein systems with end-point binding free energy calculations. Brief Bioinform. 2022;23(3):bbac127. 35395683 10.1093/bib/bbac127

[48] Xu K, Wang Z, Xiang S, Tang R, Deng Q, Ge J, Jiang Z, Yang K, Hou T, Sun H. Characterizing the cooperative effect of PROTAC systems with end-point binding free energy calculation. J Chem Inf Model. 2024;64(19):7666–7678. 39361611 10.1021/acs.jcim.4c01227

[49] Chen S-Y, Zacharias M. What makes a good protein-protein interaction stabilizer: Analysis and application of the dual-binding mechanism. ACS Cent Sci. 2023;9(5):969–979. 37252344 10.1021/acscentsci.3c00003 PMC10214505

[50] Drummond ML, Williams CI. In silico modeling of PROTAC-mediated ternary complexes: Validation and application. J Chem Inf Model. 2019;59(4):1634–1644. 30714732 10.1021/acs.jcim.8b00872

[51] Tan Z, Wortman M, Dillehay KL, Seibel WL, Evelyn CR, Smith SJ, Malkas LH, Zheng Y, Lu S, Dong Z. Small-molecule targeting of proliferating cell nuclear antigen chromatin association inhibits tumor cell growth. Mol Pharmacol. 2012;81(6):811–819. 22399488 10.1124/mol.112.077735 PMC3362894

[52] Wohlwend J, Corso G, Passaro S, Getz N, Reveiz M, Leidal K, Swiderski W, Atkinson L, Portnoi T, Chinn I, et al. Boltz-1: Democratizing biomolecular interaction modeling. bioRxiv. 2025. https://www.biorxiv.org/content/10.1101/2024.11.19.624167v4

[53] Abramson J, Adler J, Dunger J, Evans R, Green T, Pritzel A, Ronneberger O, Willmore L, Ballard AJ, Bambrick J, et al. Accurate structure prediction of biomolecular interactions with alphafold 3. Nature. 2024;630(8016):493–500. 38718835 10.1038/s41586-024-07487-w PMC11168924

[54] Chai Discovery, Boitreaud J, Dent J, McPartlon M, Meier J, Reis V, Rogozhonikov A, Wu K, Chai-1: Decoding the molecular interactions of life. bioRxiv. 2024. https://www.biorxiv.org/content/10.1101/2024.10.10.615955v2

[55] Xue F, Zhang M, Li S, Gao X, Wohlschlegel JA, Huang W, Yang Y, Deng W. SE(3)-equivariant ternary complex prediction towards target protein degradation. Nat Commun. 2025;16(1):5514. 40593782 10.1038/s41467-025-61272-5 PMC12216337

[56] Shen J, Zhou S, Che X, FKSFold: Improving AlphaFold3-type predictions of molecular glue-induced ternary complexes with Feynman-Kac-steered diffusion. bioRxiv. 2025. https://www.biorxiv.org/content/10.1101/2025.05.03.651455v1

[57] Ratni H, Scalco RS, Stephan AH. Risdiplam, the first approved small molecule splicing modifier drug as a blueprint for future transformative medicines. ACS Med Chem Lett. 2021;12(6):874–877. 34141064 10.1021/acsmedchemlett.0c00659 PMC8201486

[58] Bogden CE, Fass D, Bergman N, Nichols MD, Berger JM. The structural basis for terminator recognition by the Rho transcription termination factor. Mol Cell. 1999;3(4):487–493. 10230401 10.1016/s1097-2765(00)80476-1

[59] Wilinski D, Qiu C, Lapointe CP, Nevil M, Campbell ZT, Tanaka Hall TM, Wickens M. RNA regulatory networks diversified through curvature of the PUF protein scaffold. Nat Commun. 2015;6(1):8213. 26364903 10.1038/ncomms9213 PMC4570272

[60] Webb B, Sali A. Comparative protein structure modeling using MODELLER. Curr Protoc Bioinformatics. 2016;54(1):5–6. 10.1002/cpbi.3 PMC5031415 27322406

[61] The PyMOL molecular graphics system, version 1.2r3pre. Schrödinger, LLC (2020).

[62] Case DA, Aktulga HM, Belfon K, Ben-Shalom IY, Berryman JT, Brozell SR, Cerutti DS, Cheatham TE III, Cisneros GA, Cruzeiro VWD, et al. Amber 22. University of California, San Francisco. 2022.

[63] Gowers RJ, Linke M, Barnoud J, Reddy TJE, Melo MN, Seyler SL, Domański J, Dotson DL, Buchoux S, Kenney IM, et al. MDAnalysis: A python package for the rapid analysis of molecular dynamics simulations. In:Benthall S, Rostrup S, editors. *Proceedings of the 15th Python in Science Conference (SciPy 2016)*. Austin (TX): SciPy; 2016. p. 98–105.

[64] Michaud-Agrawal N, Denning EJ, Woolf TB, Beckstein O. MDAnalysis: A toolkit for the analysis of molecular dynamics simulations. J Comput Chem. 2011;32(10):2319–2327. 21500218 10.1002/jcc.21787 PMC3144279

[65] Virtanen P, Gommers R, Oliphant TE, Haberland M, Reddy T, Cournapeau D, Burovski E, Peterson P, Weckesser W, Bright J, et al. SciPy 1.0: Fundamental algorithms for scientific computing in Python. Nat Methods. 2020;17:261–272. 32015543 10.1038/s41592-019-0686-2 PMC7056644

[66] Duboué-Dijon E, Laage D. Characterization of the local structure in liquid water by various order parameters. J Phys Chem B. 2015;119(26):8406–8418. 26054933 10.1021/acs.jpcb.5b02936 PMC4516314

[67] Chau P-L, Hardwick AJ. A new order parameter for tetrahedral configurations. Mol Phys. 1998;93(3):511–518.

[68] Irwin JJ, Tang KG, Young J, Dandarchuluun C, Wong BR, Khurelbaatar M, Moroz YS, Mayfield J, Sayle RA. ZINC20—A free ultralarge-scale chemical database for ligand discovery. J Chem Inf Model. 2020;60(12):6065–6073. 33118813 10.1021/acs.jcim.0c00675 PMC8284596

[69] Gleeson PM, Leeson PD, van de Waterbeemd H, Physicochemical properties and compound quality. In: A. Davis, S. E. Ward, editors. *The Handbook of Medicinal Chemistry*. Cambridge (UK): Royal Society of Chemistry; 2015. Ch. 1, p. 1–31.

[70] Weininger D. SMILES, a chemical language and information system. 1. Introduction to methodology and encoding rules. J Chem Inf Comput Sci. 1988;28(1):31–36.

[71] Weininger D, Weininger A, Weininger JL, SMILES. 2. Algorithm for generation of unique SMILES notation. J Chem Inf Comput Sci. 1989;29(2):97–101.

[72] Case DA, Aktulga HM, Belfon K, Ben-Shalom IY, Berryman JT, Brozell SR, Cerutti DS, Cheatham TE III, Cisneros GA, Cruzeiro VWD, et al. Ambertools23. 2023.

[73] Le Guilloux V, Schmidtke P, Tuffery P. Fpocket: An open source platform for ligand pocket detection. BMC Bioinformatics. 2009;10(1):168. 19486540 10.1186/1471-2105-10-168 PMC2700099

[74] Schmidtke P, Bidon-Chanal A, Luque FJ, Barril X. MDpocket: Open-source cavity detection and characterization on molecular dynamics trajectories. Bioinformatics. 2011;27(23):3276–3285. 21967761 10.1093/bioinformatics/btr550

[75] Aggarwal R, Gupta A, Chelur V, Jawahar CV, Priyakumar UD. DeepPocket: Ligand binding site detection and segmentation using 3D convolutional neural networks. J Chem Inf Model. 2021;62(21):5069–5079. 34374539 10.1021/acs.jcim.1c00799

[76] Shrake A, Rupley J. Environment and exposure to solvent of protein atoms. Lysozyme and insulin. J Mol Biol. 1973;79(2):351–371. 4760134 10.1016/0022-2836(73)90011-9

[77] Santos-Martins D, Solis-Vasquez L, Tillack AF, Sanner MF, Koch A, Forli S. Accelerating AutoDock4 with GPUs and gradient-based local search. J Chem Theory Comput. 2021;17(2):1060–1073. 33403848 10.1021/acs.jctc.0c01006 PMC8063785

[78] Liu P, Agrafiotis DK, Theobald DL. Fast determination of the optimal rotational matrix for macromolecular superpositions. J Comput Chem. 2009;31(7):1561–1563. 10.1002/jcc.21439 PMC2958452 20017124

[79] Theobald DL. Rapid calculation of RMSDs using a quaternion-based characteristic polynomial. Acta Crystallogr Sect A Found Crystallogr. 2005;61(4):478–480. 10.1107/S0108767305015266 15973002

[80] Still WC, Tempczyk A, Hawley RC, Hendrickson T. Semianalytical treatment of solvation for molecular mechanics and dynamics. J Am Chem Soc. 1990;112(16):6127–6129.

[81] Kollman PA, Massova I, Reyes C, Kuhn B, Huo S, Chong L, Lee M, Lee T, Duan Y, Wang W, et al. Calculating structures and free energies of complex molecules: Combining molecular mechanics and continuum models. Acc Chem Res. 2000;33(12):889–897. 11123888 10.1021/ar000033j

[82] Onufriev A, Bashford D, Case DA. Exploring protein native states and large-scale conformational changes with a modified generalized born model. Proteins Struct Funct Bioinf. 2004;55(2):383–394. 10.1002/prot.20033 15048829

[83] Bolcato G, Heid E, Boström J. On the value of using 3D shape and electrostatic similarities in deep generative methods. J Chem Inf Model. 2022;62(6):1388–1398. 35271260 10.1021/acs.jcim.1c01535 PMC8965872

[84] Landrum G, Tosco P, Kelley B, Ric, Cosgrove D, Sriniker, Gedeck, Vianello R, Schneider N, Kawashima E, et al. Strets123, rdkit/rdkit: 2023_09_4(q3 2023) release. 2024.

[85] Pedregosa F, Varoquaux G, Gramfort A, Michel V, Thirion B, Grisel O, Blondel M, Prettenhofer P, Weiss R, Dubourg V, et al. Scikit-learn: Machine learning in Python. J Mach Learn Res. 2011;12:2825–2830.

[86] Chawla NV, Bowyer KW, Hall LO, Kegelmeyer WP. SMOTE: Synthetic minority over-sampling technique. J Artif Intell Res. 2002;16:321–357.

[87] Brodersen KH, Ong CS, Stephan KE, Buhmann JM. The balanced accuracy and its posterior distribution. In: 2010 20th International Conference on Pattern Recognition. IEEE Computer Society, USA; 2010. p. 3121–3124.

[88] Baldi P, Brunak S, Chauvin Y, Andersen CAF, Nielsen H. Assessing the accuracy of prediction algorithms for classification: An overview. Bioinformatics. 2000;16(5):412–424. 10871264 10.1093/bioinformatics/16.5.412

[89] Fawcett T. An introduction to ROC analysis. Pattern Recogn Lett. 2006;27(8):861–874.

[90] Van Rossum G, Drake FL. Python 3 reference manual. Scotts Valley (CA): CreateSpace; 2009.

[91] Lemaître G, Nogueira F, Aridas CK. Imbalanced-learn: A python toolbox to tackle the curse of imbalanced datasets in machine learning. J Mach Learn Res. 2017;18(17):1–5.

[92] Harris CR, Millman KJ, van der Walt SJ, Gommers R, Virtanen P, Cournapeau D, Wieser E, Taylor J, Berg S, Smith NJ, et al. Array programming with NumPy. Nature. 2020;585(7825):357–362. 32939066 10.1038/s41586-020-2649-2 PMC7759461

[93] McKinney W. Data structures for statistical computing in Python. In: van der Walt S, Millman J, editors. *Proceedings of the 9th Python in Science Conference*. Austin (TX): SciPy; 2010. p. 56–61.

[94] Rogers D, Hahn M. Extended-connectivity fingerprints. J Chem Inf Model. 2010;50(5):742–754. 20426451 10.1021/ci100050t

[95] Degen J, Wegscheid-Gerlach C, Zaliani A, Rarey M. On the art of compiling and using drug-like chemical fragment spaces. ChemMedChem. 2008;3(10):1503–1507. 18792903 10.1002/cmdc.200800178

[96] Vasilyev N, Polonskaia A, Darnell JC, Darnell RB, Patel DJ, Serganov A. Crystal structure reveals specific recognition of a G-quadruplex RNA by a β-turn in the RGG motif of FMRP. Proc Natl Acad Sci USA. 2015;112(39):E5391–E5400. 26374839 10.1073/pnas.1515737112 PMC4593078

[97] Goto-Ito S, Ito T, Kuratani M, Bessho Y, Yokoyama S. Tertiary structure checkpoint at anticodon loop modification in tRNA functional maturation. Nat Struct Mol Biol. 2009;16(10):1109–1115. 19749755 10.1038/nsmb.1653

[98] Osawa T, Kimura S, Terasaka N, Inanaga H, Suzuki T, Numata T. Structural basis of tRNA agmatinylation essential for AUA codon decoding. Nat Struct Mol Biol. 2011;18(11):1275–1280. 22002223 10.1038/nsmb.2144

[99] Thomas SR, Keller CA, Szyk A, Cannon JR, LaRonde-LeBlanc NA. Structural insight into the functional mechanism of Nep1/Emg1 N1-specific pseudouridine methyltransferase in ribosome biogenesis. Nucleic Acids Res. 2010;39(6):2445–2457. 21087996 10.1093/nar/gkq1131 PMC3064781

[100] Jiang Y, Yu H, Li F, Cheng L, Zhu L, Shi Y, Gong Q. Unveiling the structural features that determine the dual methyltransferase activities of streptococcus pneumoniae RlmCD. PLOS Pathog. 2018;14(11): Article e1007379. 30388185 10.1371/journal.ppat.1007379 PMC6235398

[101] Ryu H, Grove TL, Almo SC, Kim J. Identification of a novel tRNA wobble uridine modifying activity in the biosynthesis of 5-methoxyuridine. Nucleic Acids Res. 2018;46(17):9160–9169. 29982645 10.1093/nar/gky592 PMC6158493

[102] Esakova OA, Grove TL, Yennawar NH, Arcinas AJ, Wang B, Krebs C, Almo SC, Booker SJ. Structural basis for tRNA methylthiolation by the radical SAM enzyme MiaB. Nature. 2021;597(7877):566–570. 34526715 10.1038/s41586-021-03904-6 PMC9107155

[103] Betat H, Mörl M. The CCA-adding enzyme: A central scrutinizer in tRNA quality control. BioEssays. 2015;37(9):975–982. 26172425 10.1002/bies.201500043

[104] Goyal N, Chandra A, Qamar I, Singh N. Structural studies on dihydrouridine synthase aA (DusA) from pseudomonas aeruginosa. Int J Biol Macromol. 2019;132:254–264. 30928375 10.1016/j.ijbiomac.2019.03.209

[105] Li L, Ye K. Crystal structure of an H/ACA box ribonucleoprotein particle. Nature. 2006;443(7109):302–307. 16943774 10.1038/nature05151

[106] Škrinjar P, Eberhardt J, Tauriello G, Schwede T, Durairaj J, Have protein-ligand cofolding methods moved beyond memorisation? bioRxiv. 2025. https://www.biorxiv.org/content/10.1101/2025.02.03.636309v1 10.1038/s41594-026-01797-5 PMC13661253 42103936

[107] Berg JM, Tymoczko JL, Stryer L. Biochemistry. 6th ed. New York (NY): W.H. Freeman; 2007.

[108] Yang L, Wang C, Li F, Zhang J, Nayab A, Wu J, Shi Y, Gong Q. The human RNA-binding protein and E3 ligase MEX-3C binds the MEX-3-recognition element (MRE) motif with high affinity. J Biol Chem. 2017;292(39):16221–16234. 28808060 10.1074/jbc.M117.797746 PMC5625052

[109] Yang Y, Eichhorn CD, Wang Y, Cascio D, Feigon J. Structural basis of 7SK RNA 5′-gamma-phosphate methylation and retention by MePCE. Nat Chem Biol. 2018;15(2):132–140. 30559425 10.1038/s41589-018-0188-z PMC6339579

[110] Price SR, Evans PR, Nagai K. Crystal structure of the spliceosomal U2″-U2A′ protein complex bound to a fragment of U2 small nuclear RNA. Nature. 1998;394(6694):645–650. 9716128 10.1038/29234

[111] Tanikawa M, Sanjiv K, Helleday T, Herr P, Mortusewicz O. The spliceosome U2 snRNP factors promote genome stability through distinct mechanisms; transcription of repair factors and R-loop processing. Oncogene. 2016;5(12):e280. 10.1038/oncsis.2016.70 PMC5177769 27991914

[112] Abasi M, Bazi Z, Mohammadi-Yeganeh S, Soleimani M, Haghpanah V, Zargami N, Ghanbarian H. 7SK small nuclear RNA transcription level down-regulates in human tumors and stem cells. Med Oncol. 2016;33(11):128. 27752877 10.1007/s12032-016-0841-x

[113] Trott O, Olson AJ. Autodock Vina: Improving the speed and accuracy of docking with a new scoring function, efficient optimization, and multithreading. J Comput Chem. 2009;31(2):455–461. 10.1002/jcc.21334 PMC3041641 19499576

[114] Eberhardt J, Santos-Martins D, Tillack AF, Forli S. AutoDock Vina 1.2.0: New docking methods, expanded force field, and Python bindings. J Chem Inf Model. 2021;61(8):3891–3898. 34278794 10.1021/acs.jcim.1c00203 PMC10683950

[115] Tomita K, Ishitani R, Fukai S, Nureki O. Complete crystallographic analysis of the dynamics of CCA sequence addition. Nature. 2006;443(7114):956–960. 17051158 10.1038/nature05204

[116] Pellegrini O, Li de la Sierra-Gallay I, Piton J, Gilet L, Condon C. Activation of tRNA maturation by downstream uracil residues in B. subtilis. Structure. 2012;20(10):1769–1777. 22940585 10.1016/j.str.2012.08.002

[117] Byrne RT, Jenkins HT, Peters DT, Whelan F, Stowell J, Aziz N, Kasatsky P, Rodnina MV, Koonin EV, Konevega AL, et al. Major reorientation of tRNA substrates defines specificity of dihydrouridine synthases. Proc Natl Acad Sci USA. 2015;112(19):6033–6037. 25902496 10.1073/pnas.1500161112 PMC4434734

[118] Liu X, Shen S, Wu P, Li F, Liu X, Wang C, Gong Q, Wu J, Yao X, Zhang H, et al. Structural insights into dimethylation of 12S rRNA by TFB1M: Indispensable role in translation of mitochondrial genes and mitochondrial function. Nucleic Acids Res. 2019;47(14):7648–7665. 31251801 10.1093/nar/gkz505 PMC6698656

[119] Yang Y, Harris KA, Widner DL, Breaker RR. Structure of a bacterial OapB protein with its OLE RNA target gives insights into the architecture of the OLE ribonucleoprotein complex. Proc Natl Acad Sci USA. 2021;118(9): Article e2020393118. 33619097 10.1073/pnas.2020393118 PMC7936274

