# Supplementary material for: In Silico Analysis of Potential Stabilizer Binding Sites at Protein–RNA Interfaces
Source: Comput Struct Biotechnol J. 2026 Mar 31;35(1):0016. doi: 10.34133/csbj.0016 (PMC13082465; doi:10.34133/csbj.0016)
Supplement: Supplementary 1 — Notes S1 to S4 Figs. S1 to S14 Table S1 [file csbj.0016.f1.pdf]

Supporting Information: In Silico Analysis of Potential Stabilizer Binding Sites at Protein-RNA Interfaces

L. Vollmers[a,1,*], S.-Y. Chen[b,2], M. Zacharias[a,3]

[a] *Center for functional Protein Assemblies, Technical University of Munich, Germany*
[b] *Department of Chemistry and Applied Biosciences, ETH Zurich, Switzerland*

Abstract

Stabilizing rather than interfering with protein-protein interactions has emerged as a promising concept for designing drug molecules that can modulate protein-protein interactions. In addition to protein-protein interactions, protein-RNA interaction are involved in numerous cellular metabolic and regulatory processes, and it is desirable to stabilize these interactions, e.g., for potential pharmaceutical applications. In order to assess the potential that lies in protein-RNA interaction modulation via stabilizing drug molecules, we analyzed the binding interfaces of a large set of 87 protein-RNA complexes. Our *in silico* analysis revealed many interface cavities and potential binding sites that could fit drug-like compounds that can simultaneously interact with the protein and RNA. Through a systematic workflow combining interface pocket detection, molecular docking, and MMGB/SA calculations, we evaluated multiple pocket detection algorithms and identified key chemical features shared among *in silico* stabilizer candidates. The docking approach successfully reproduced portions of known experimental ligands and could be valuable for suggesting potential stabilizers of protein-RNA interactions.

[*] Corresponding author

Email addresses: luis.vollmers@tum.de (L. Vollmers), shu-yu.chen@ethz.ch (S.-Y. Chen), zacharias@tum.de (M. Zacharias)

[1] This is the first author footnote.

[2] This is the second author footnote.

[3] This is the third author footnote.

S1. Setup of Random Forest Modeling

For training the random forest model classifier, 77 features were compiled for each pocket. These features are itemized below. The relative water density denotes the division of the pocket water density by bulk water density, and the features from docking statistics are calculated from the docked compounds.

Features Collected From Apo-Complex MD Simulations:

- Pocket Volume in $Å^3$ [mean, std, min, Q1, Q2, Q3, max]
- Pocket Water Density in $Å^{-3}$ [mean, std, min, Q1, Q2, Q3, max]
- relative Pocket Water Density [mean, std, min, Q1, Q2, Q3, max]
- Translational Order Parameter S_k: [mean, std, min, Q1, Q2, Q3, max]

Features Collected From Docking Statistics:

- Docking Score: [mean, std, min, Q1, Q2, Q3, max]
- BSA rank 1 (BSA1): [mean, std, min, Q1, Q2, Q3, max]
- BSA rank 2 (BSA2): [mean, std, min, Q1, Q2, Q3, max]
- BSA1 / BSA2: [mean, std, min, Q1, Q2, Q3, max]
- BSA1 - BSA2: [mean, std, min, Q1, Q2, Q3, max]
- 1 - BSA2 / BSA1: [mean, std, min, Q1, Q2, Q3, max]
- BSA1/BSA2 - 1: [mean, std, min, Q1, Q2, Q3, max]

Each pocket equates to one data row; thus, the complete data table is 260 rows and 77 columns with a positive-to-negative class ratio of 0.54 for basic stabilizer candidates and 0.21 for elevated stabilizer candidates.
The pre-processing grid search for the random forest classification focuses on data handling and class imbalance. Class imbalance in the training data was mitigated by employing a two-step resampling approach using the imbalanced-learn Python library. First, the minority class was oversampled using synthetic minority over-sampling technique (SMOTE)[1] to achieve target positive-to-negative class ratios of 0.6, 0.65, and 0.7. The SMOTE algorithm generated synthetic samples by interpolating feature values between existing minority-class instances and their k-nearest neighbors, with k values

of 5, 10, and 20. Following oversampling, a random undersampling strategy was applied to the majority class to further balance the dataset, targeting the positive-to-negative class ratios of 0.7, 0.8, and 0.9. Model hyperparameters were optimized using scikit-learn's HalvingRandomSearchCV[2]. The outer validation employed a 5-fold stratified cross validation (CV), while the inner optimization utilized a shuffle-split cross-validation with configurable test sizes of 15%, 10%, 9%, 8%, and 7% of the data. For each parameter configuration, candidate values included:

- Decision tree splitting criteria: gini impurity, entropy, and log-loss.
- Maximum tree depth: values between 10, 20, and unlimited depth.
- Minimum samples for node splitting: 4-13 samples.
- Minimum samples per leaf: 1-13 samples.
- Feature selection strategy: log2, square root, all features, or fixed numbers (10-50).
- Number of estimator trees: 100-700.
- Class weight schemes: none, balanced, or balanced subsample.

Five hundred candidate configurations were evaluated with a factor-3 successive halving approach for each hyperparameter optimization run, retaining the most promising third of configurations at each iteration. A nested cross-validation approach was implemented to provide robust performance estimates while preventing data leakage. Aggregated metrics (combining predictions across all folds) and fold-wise metrics were calculated to evaluate overall performance and consistency.

In summary, the different pre-processing parameters of the grid search resulted in a total of 135 5-fold nested CV runs with hyperparameter tuning. For reproducibility, a consistent random seed was maintained throughout the pipeline. The hyperparameter search, model training, and evaluation process generated comprehensive output files documenting performance metrics, feature importance rankings, and serialized model objects for subsequent analysis or deployment.

The feature importance measured by the impurity decrease in percent is only meaningful if the training resulted in a predictive model. The performance metrics collected from all trained models indicate that our random forest classifiers are informative predictors. When predicting the absence or presence of a basic stabilizer candidate, our models reach aggregated ROC area under

the curve (ROC AUC) values up to 0.793 (receiver operator characteristic (ROC) curves are shown in figure S11A). From the 135 pre-processing grid search runs, the lowest combined ROC AUC still reaches 0.722. The more important sensitivity reaches values up to 0.728, indicating that around 73 % of basic stabilizer candidates could be successfully predicted by the classifier. The metrics reveal slightly inferior predictiveness for the elevated stabilizer candidates, which might be caused by the higher class imbalance. Here, the combined ROC AUC reaches up to 0.773 for the highest scoring grid search run and 0.684 for the lowest. The model reaches sensitivity values of up to 0.609 and decreases until 0.391 for the least performant hyperparameter combination (ROC curves are shown in figure S11B).

S2. Binding Affinities and Comparison to Experiment

Table S1: All PDB entries of investigated protein-RNA complexes listed alphabetically.[3, 4, 5, 6, 7, 8, 9, 10, 11, 12, 13, 14, 15, 16, 17, 18, 19, 20, 21, 22, 23, 24, 25, 26, 27, 28, 29, 30, 31, 32, 33, 34, 34, 35, 36, 37, 38, 39, 40, 41, 42, 43, 44, 45, 46, 47, 48, 49, 50, 51, 52, 53, 54, 55, 56, 57, 58, 59, 59, 60, 61, 62, 63, 64, 65, 66, 67, 68, 69, 70, 71, 72, 73, 74, 75, 76, 77, 78, 79, 80, 81, 82, 83, 84, 85, 86, 87]

List of All Investigated PDB entries										
1A9N	1C0A	1DFU	1DK1	1DUL	1E7K	1EC6	1F7Y	1HC8	1I6U	1JBR
1JID	1K8W	1KUQ	1MMS	1MZP	1OOA	1QA6	1R9F	1RLG	1S03	1URN
1Y39	1ZE2	1ZH5	1ZL3	1ZSE	2ANN	2DRB	2F8K	2HVY	2HW8	2PJP
2PLY	2XDB	2Y8W	2ZZM	3AMT	3BT7	3IAB	3OIN	3U4M	4AL5	4BW0
4C8Y	4GCW	4L8H	4LGT	4PDB	4QIL	4R8I	4TUW	4YCP	4YVJ	4ZLD
5A0X	5DEA	5D04	5F5H	5UD5	5V0E	5WWE	5WWW	5ZQ0	5ZW4	6AAX
6CF2	6CMN	6DCB	6DU4	6F4G	6FQ3	6H9H	6JVX	6LAS	6PPV	6SX0
6SY4	6TQB	6XH2	6XKI	6YYM	7K9D	7MJV	7OZQ	7P0V	7WKP	

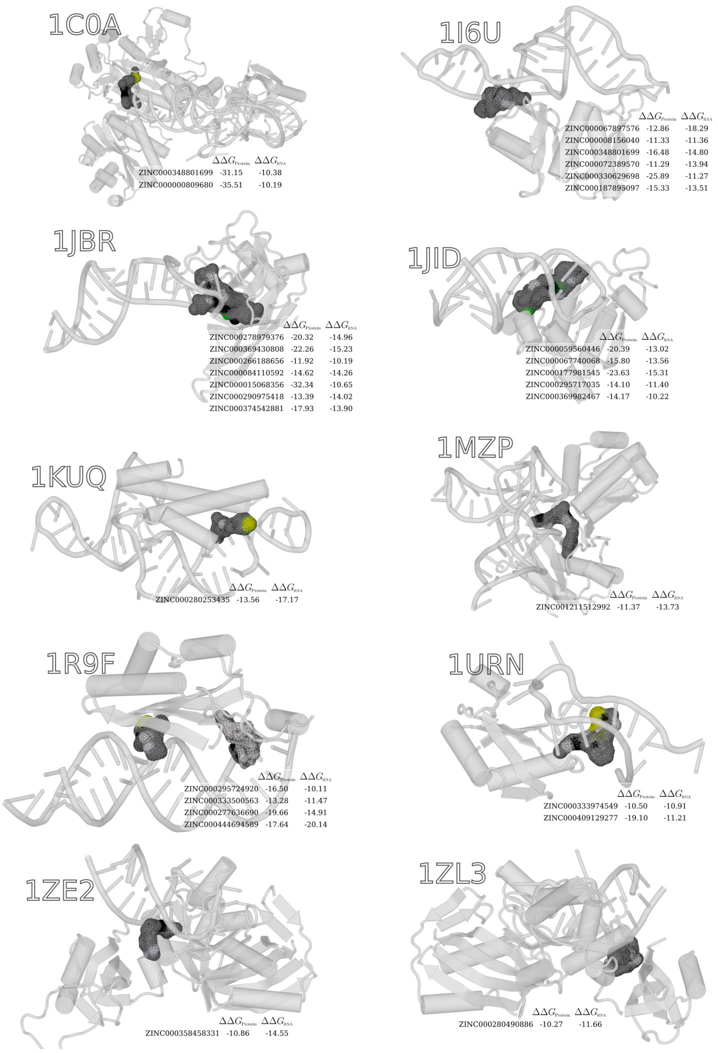

Figure S1: Overview of the 34 complexes with at least one elevated stabilizer. The protein and RNA molecules are shown in transparent grey, with their PDB IDs printed in the upper-left corner of each complex. The variable number of stabilizer candidates is shown as overlapping wireframes. The MMGBSA derived binding free energies of the ligand with the protein and the RNA are tabulated in $\text{kcal}\,\text{mol}^{-1}$ with the ZINC ID of each organic ligand. The remaining complexes are shown in figures S2 and S3.

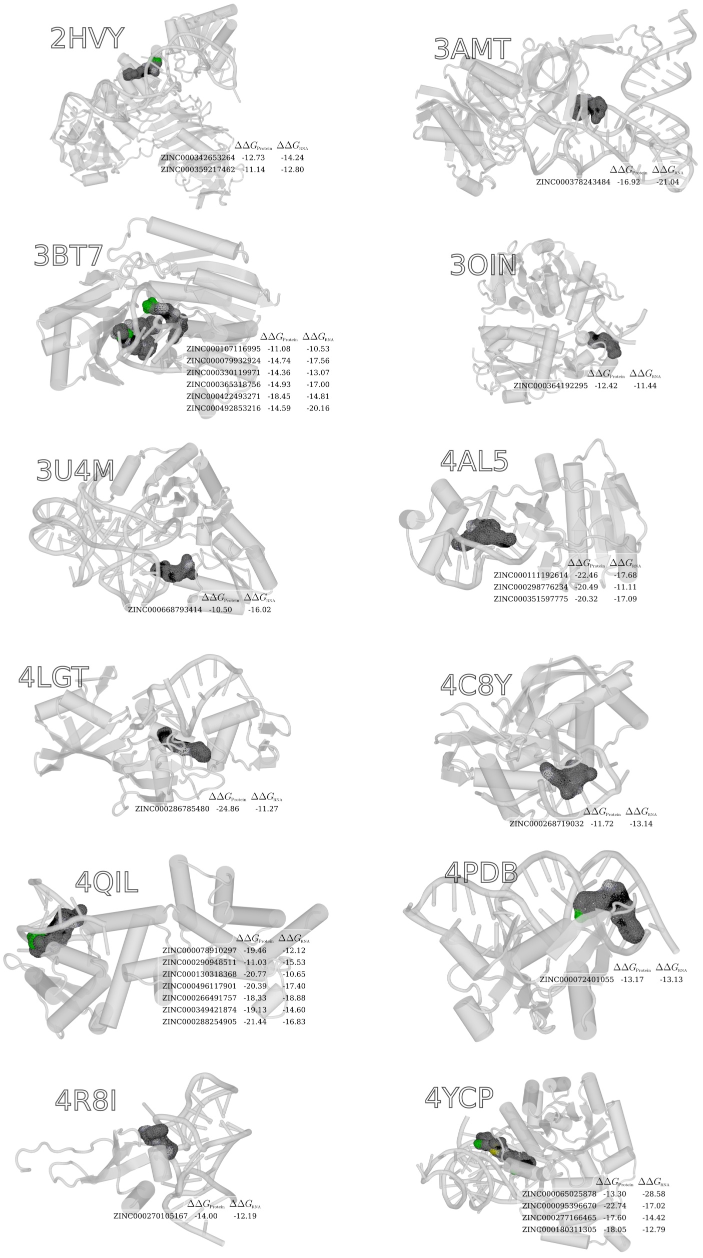

Figure S2: Continuation of S1. Energies in $\text{kcal}\,\text{mol}^{-1}$

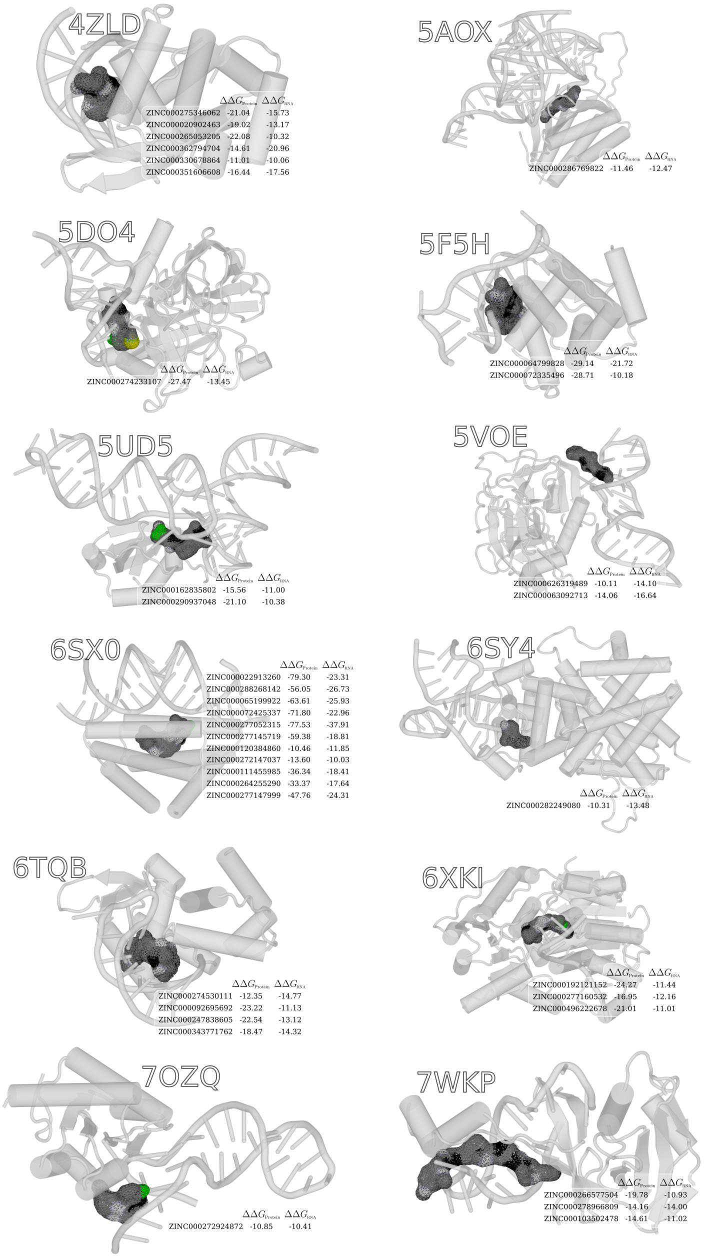

Figure S3: Continuation of S2. Energies in $\mathrm{kcal\,mol^{-1}}$

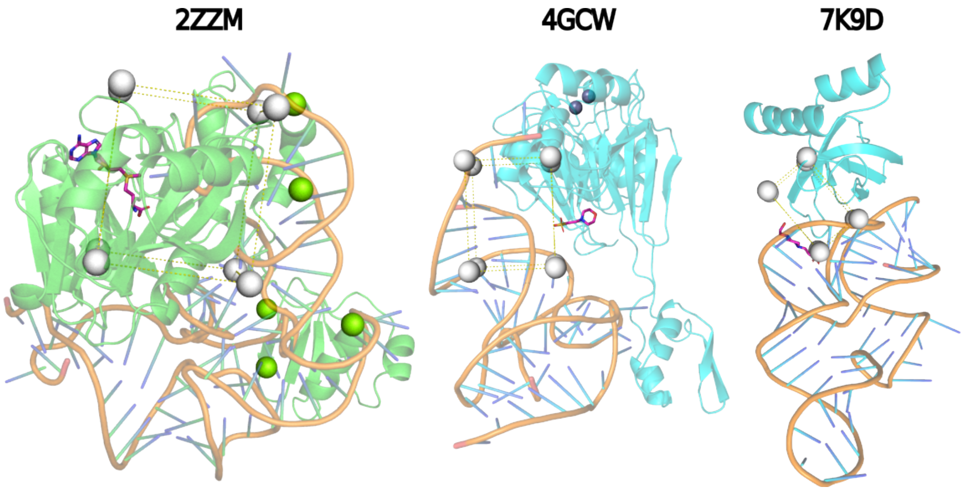

Figure S4: Complexes with experimentally resolved binders whose pockets were not matched by the applied workflow. The protein and RNA macromolecules are depicted in cartoon format, and the binding compound is shown in pink licorice. The white spheres and the yellow dotted vertices mark the cuboid volumes generated for large-scale docking. Although half of the SAM molecule reaches into the pocket cuboid for 2ZZM, the substantial mismatch between pocket and ligand placement impedes perspective docking attempts to match the experimental ligand. Meanwhile, for the other two, the HEPES and MES are outside the cuboid boundary.

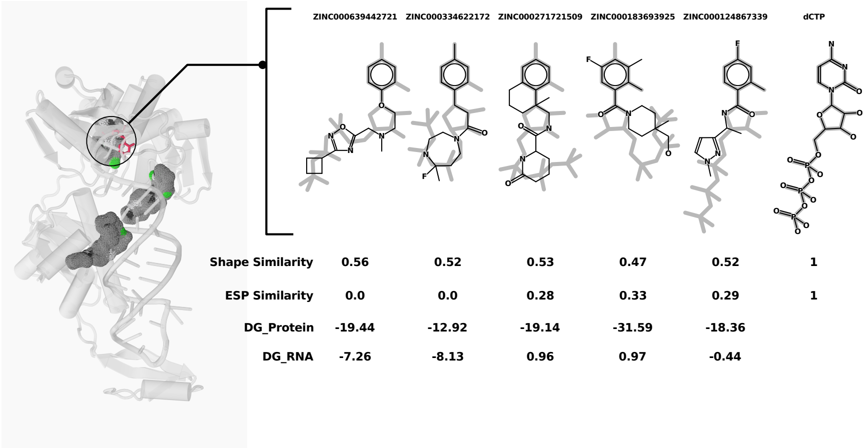

	ZINC000639442721	ZINC000334622172	ZINC000271721509	ZINC000183693925	ZINC000124867339	dCTP
Shape Similarity	0.56	0.52	0.53	0.47	0.52	1
ESP Similarity	0.0	0.0	0.28	0.33	0.29	1
DG_Protein	-19.44	-12.92	-19.14	-31.59	-18.36	
DG_RNA	-7.26	-8.13	0.96	0.97	-0.44	

Figure S5: 2DRB: Overview of *in silico* ligands binding to the experimental binding site.[88] The Macromolecules are shown as glassy cartoon with bound *in silico* ligands shown as wireframes and the experimental ligand shown as red licorice. The ligands at the experimental binding site are detailed as black skeletal formulas with the experimental gray scaffold in the background as visual guidance. The experimental ligand's black skeletal formula is shown on the far right. MMGBSA energies to protein and RNA, Shape and ESP similarities from espsim are listed below.[89, 90]

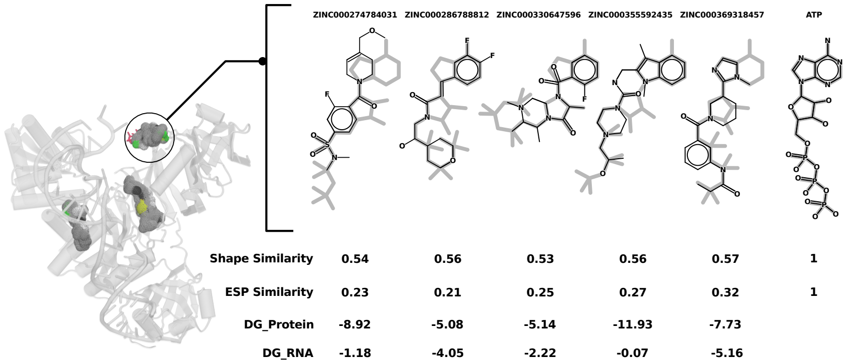

	ZINC000274784031	ZINC000286788812	ZINC000330647596	ZINC000355592435	ZINC000369318457	ATP
Shape Similarity	0.54	0.56	0.53	0.56	0.57	1
ESP Similarity	0.23	0.21	0.25	0.27	0.32	1
DG_Protein	-8.92	-5.08	-5.14	-11.93	-7.73	
DG_RNA	-1.18	-4.05	-2.22	-0.07	-5.16	

Figure S6: 2HVY: Overview of *in silico* ligands binding to the experimental binding site.[32] The Macromolecules are shown as glassy cartoon with bound *in silico* ligands shown as wireframes and the experimental ligand shown as red licorice. The ligands at the experimental binding site are detailed as black skeletal formulas with the experimental gray scaffold in the background as visual guidance. The experimental ligand's black skeletal formula is shown on the far right. MMGBSA energies to protein and RNA, Shape and ESP similarities from espsim are listed below.[89, 90]

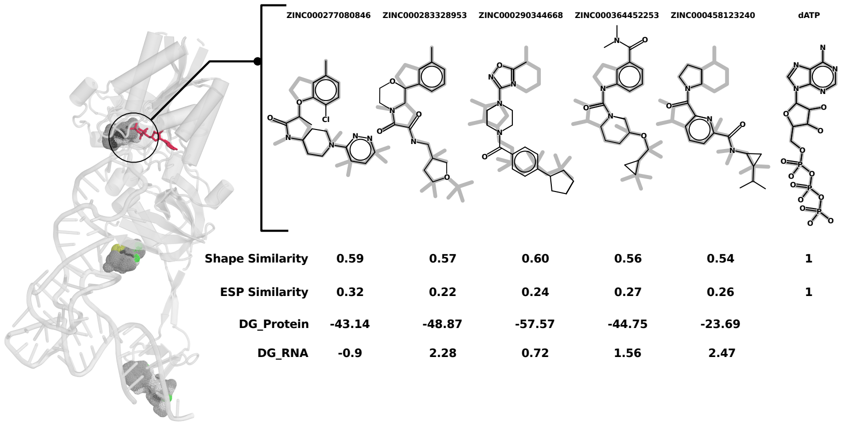

	ZINC000277080846	ZINC000283328953	ZINC000290344668	ZINC000364452253	ZINC000458123240	dATP
Shape Similarity	0.59	0.57	0.60	0.56	0.54	1
ESP Similarity	0.32	0.22	0.24	0.27	0.26	1
DG_Protein	-43.14	-48.87	-57.57	-44.75	-23.69	
DG_RNA	-0.9	2.28	0.72	1.56	2.47	

Figure S7: 3AMT: Overview of *in silico* ligands binding to the experimental binding site.[38] The Macromolecules are shown as glassy cartoon with bound *in silico* ligands shown as wireframes and the experimental ligand shown as red licorice. The ligands at the experimental binding site are detailed as black skeletal formulas with the experimental gray scaffold in the background as visual guidance. The experimental ligand's black skeletal formula is shown on the far right. MMGBSA energies to protein and RNA, Shape and ESP similarities from espsim are listed below.[89, 90]

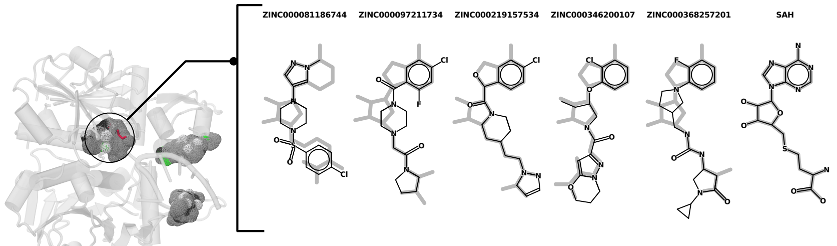

	ZINC000081186744	ZINC000097211734	ZINC000219157534	ZINC000346200107	ZINC000368257201	SAH
Shape Similarity	0.61	0.60	0.59	0.65	0.59	1
ESP Similarity	0.48	0.60	0.62	0.60	0.66	1
DG_Protein	-64.35	-45.56	-35.47	-17.19	-40.15	-39.24
DG_RNA	0.35	-0.95	-1.87	-2.27	-1.36	-0.34

Figure S8: 3OIN: Overview of *in silico* ligands binding to the experimental binding site.[41] The Macromolecules are shown as glassy cartoon with bound *in silico* ligands shown as wireframes and the experimental ligand shown as red licorice. The ligands at the experimental binding site are detailed as black skeletal formulas with the experimental gray scaffold in the background as visual guidance. The experimental ligand's black skeletal formula is shown on the far right. MMGBSA energies to protein and RNA, Shape and ESP similarities from espsim are listed below.[89, 90]

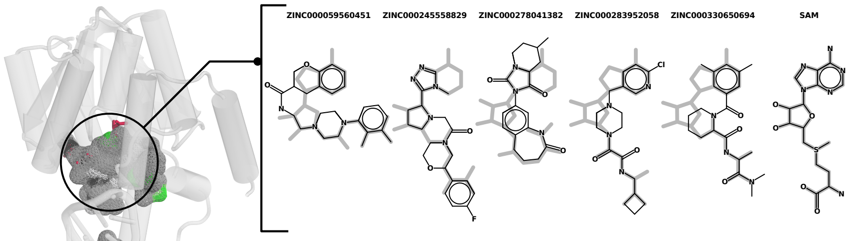

	ZINC000059560451	ZINC000245558829	ZINC000278041382	ZINC000283952058	ZINC000330650694	SAM
Shape Similarity	0.61	0.61	0.58	0.60	0.60	1
ESP Similarity	0.15	0.24	0.29	0.18	0.26	1
DG_Protein	-38.71	-29.37	-18.21	-48.83	-14.56	-78.23
DG_RNA	-5.85	2.75	-4.13	1.13	3.05	2.25

Figure S9: 5ZW4: Overview of *in silico* ligands binding to the experimental binding site.[65] The Macromolecules are shown as glassy cartoon with bound *in silico* ligands shown as wireframes and the experimental ligand shown as red licorice. The ligands at the experimental binding site are detailed as black skeletal formulas with the experimental gray scaffold in the background as visual guidance. The experimental ligand's black skeletal formula is shown on the far right. MMGBSA energies to protein and RNA, Shape and ESP similarities from espsim are listed below.[89, 90]

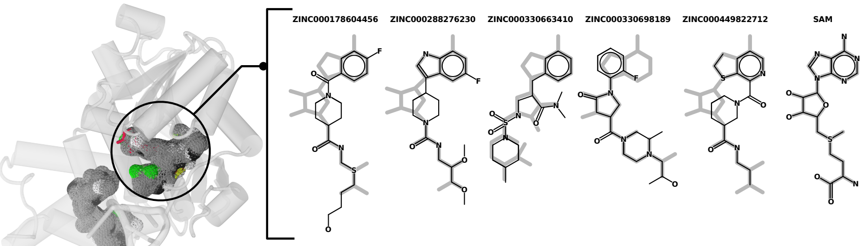

	ZINC000178604456	ZINC000288276230	ZINC000330663410	ZINC000330698189	ZINC000449822712	SAM
Shape Similarity	0.56	0.62	0.55	0.64	0.56	1
ESP Similarity	0.27	0.23	0.24	0.21	0.22	1
DG_Protein	-26.81	-39.14	-41.35	-39.97	-38.5	-43.93
DG_RNA	-1.41	-2.13	-0.93	-2.71	-2.19	0.66

Figure S10: 6AAX: Overview of *in silico* ligands binding to the experimental binding site.[66] The macromolecules are shown as glassy cartoon with bound *in silico* ligands shown as wireframes and the experimental ligand shown as red licorice. The ligands at the experimental binding site are detailed as black skeletal formulas with the experimental gray scaffold in the background as visual guidance. The experimental ligand's black skeletal formula is shown on the far right. MMGBSA energies to protein and RNA, Shape and ESP similarities from espsim are listed below.[89, 90]

S3. Results from Random Forest Modeling and Feature Importance Analysis

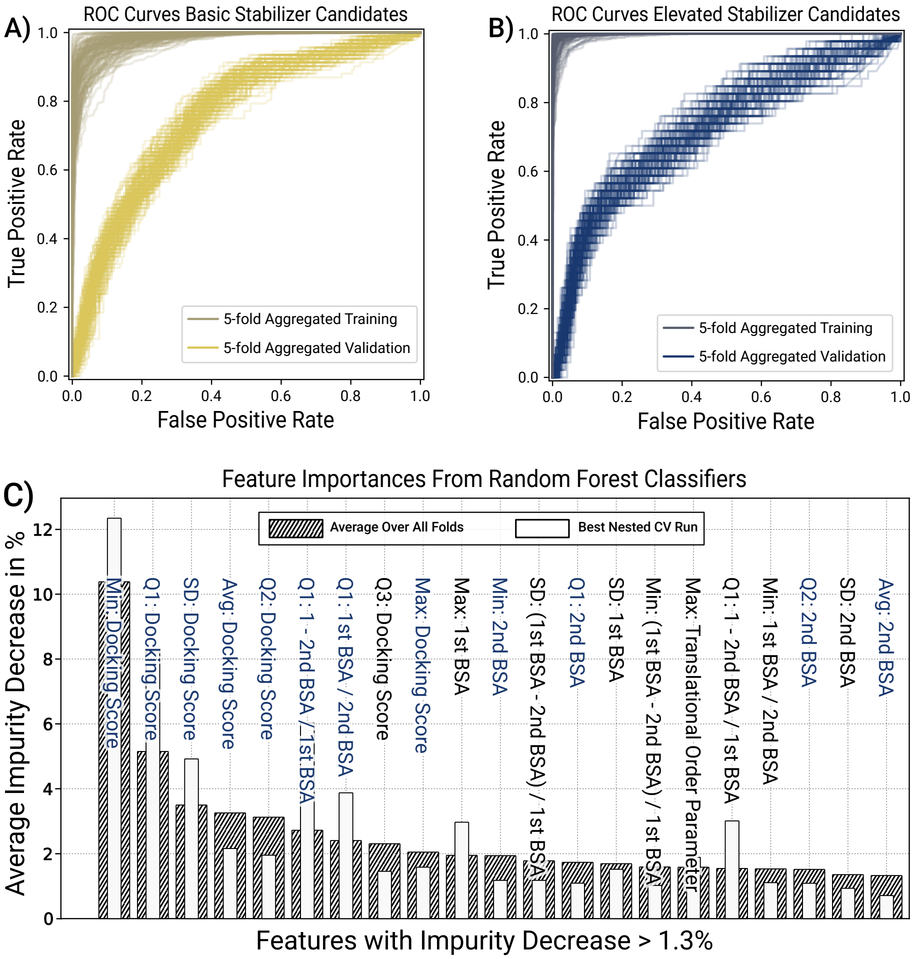

Figure S11: **A)** ROC curves of all pre-processing grid searches with the presence of basic stabilizer candidates and **B)** elevated stabilizer candidates. Each curve depicts the aggregated ROC curve of the 5-fold outer CV, after absolving hyperparameter tuning using halving random grid search. **B)** Feature importance results from the random forest classifier. The shaded bars depict the average results across pre-processing grid iterations, while the white bars show the loop that achieves the highest balanced accuracy. The feature names are shown next to the bars. Important features overlapping with the modeling of basic stabilizer candidates are labeled in blue.

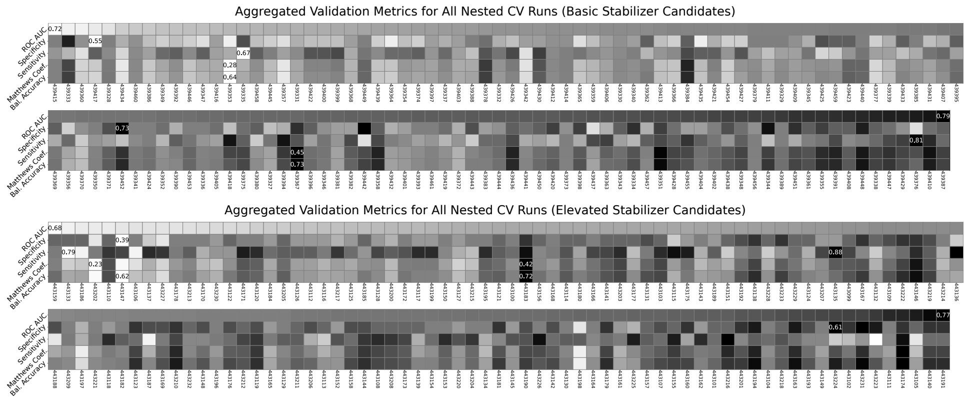

Figure S12: The evaluation metrics from the random forest classifier runs with the performances for targeting basic stabilizer candidates on top and elevated stabilizer candidates below. The aggregated values for each pre-processing grid search are reported for the ROC AUC, Specificity, Sensitivity, Matthews correlation coefficient, and balanced accuracy. Each run ID is shown on the x-axis for in-house processing. The runs are sorted by ascending ROC AUC values, with light tiles indicating weak performance and dark tiles indicating strong performance in the 5-fold grid search.

S4. Chemical Fragmentation Analysis

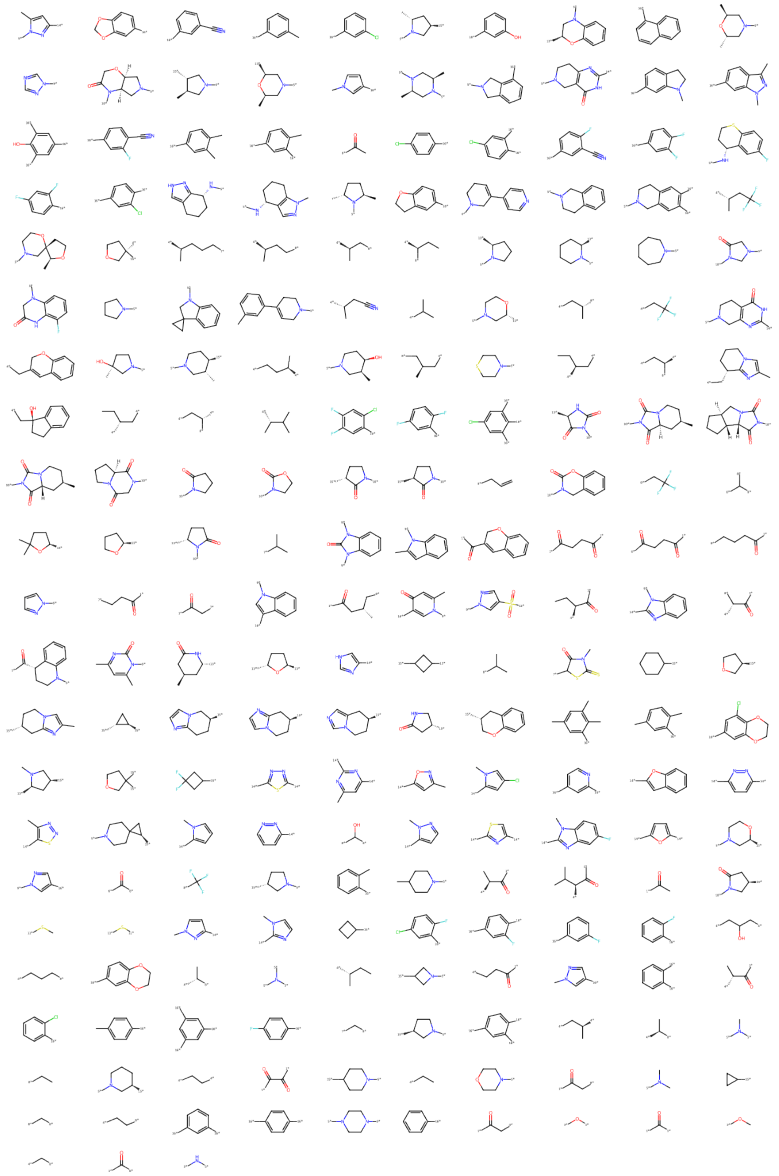

Figure S13: Complete set of BRICS fragments generated from the elevated stabilizer candidates. In total, it is 213 fragments that can be combined following rules laid out elsewhere to generate novel compounds.[91]

S5. Comparison to Chai-1 Predictions

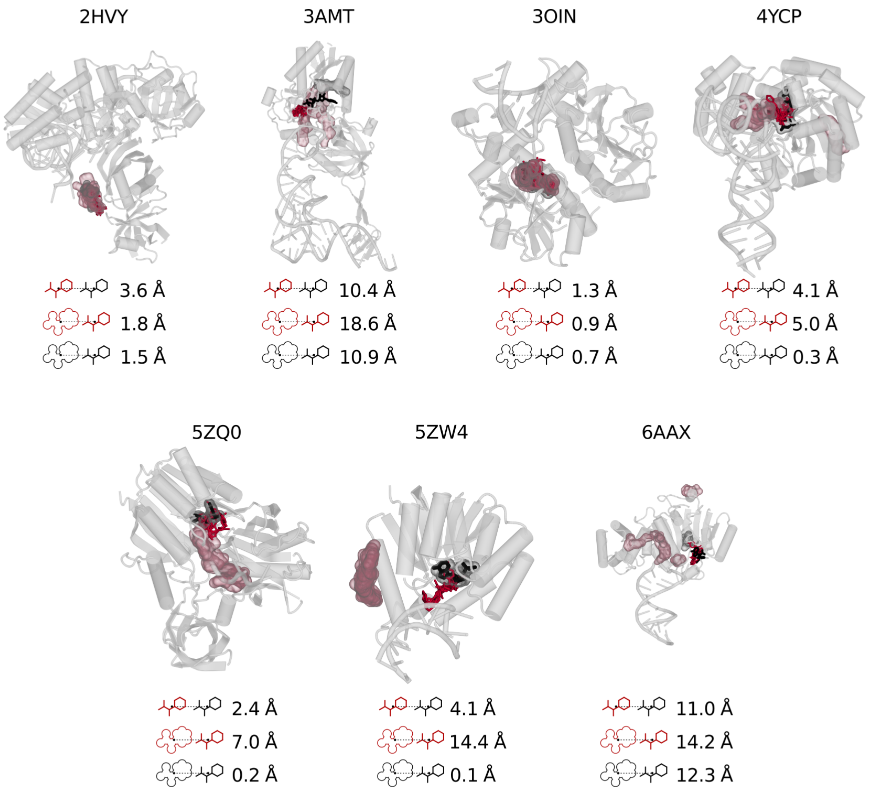

Figure S14: Visualization of the experimental complexes 2HVY, 3AMT, 3OIN, 4YCP, 5ZQ0, 5ZW4 and 6AAX. The images contain the experimental ligand in black licorice representation, the *in silico* ligands in red licorice representation, and the Chai-1 placements as transparent surface representation in the respective color. Below each render, the COM distances are given, with the icons closely resembling each representation. The renders for 2ZZM, 4GCW, and 7K9D are omitted because the outlined workflow did not identify their pockets.

References

[1] N. V. Chawla, K. W. Bowyer, L. O. Hall, W. P. Kegelmeyer, SMOTE: Synthetic minority over-sampling technique, J. Artif. Intell. Res. 16 (2002) 321–357. doi:10.1613/jair.953.
URL http://dx.doi.org/10.1613/jair.953

[2] F. Pedregosa, G. Varoquaux, A. Gramfort, V. Michel, B. Thirion, O. Grisel, M. Blondel, P. Prettenhofer, R. Weiss, V. Dubourg, J. Vanderplas, A. Passos, D. Cournapeau, M. Brucher, M. Perrot, E. Duchesnay, Scikit-learn: Machine learning in Python, J. Mach. Learn. Res. 12 (2011) 2825–2830.

[3] S. Eiler, A.-C. Dock-Bregeon, L. Moulinier, J.-C. Thierry, D. Moras, Synthesis of aspartyl-tRNAAsp in escherichia coli—a snapshot of the second step, The EMBO Journal 18 (22) (1999) 6532–6541. doi:10.1093/emboj/18.22.6532.
URL http://dx.doi.org/10.1093/emboj/18.22.6532

[4] M. Lu, T. A. Steitz, Structure of escherichia coli ribosomal protein L25 complexed with a 5S rRNA fragment at 1.8-å resolution, Proceedings of the National Academy of Sciences 97 (5) (2000) 2023–2028. doi:10.1073/pnas.97.5.2023.
URL http://dx.doi.org/10.1073/pnas.97.5.2023

[5] P. Dumas, A. Nikulin, A. Serganov, E. Ennifar, S. Tishchenko, N. Nevskaya, W. Shepard, C. Portier, M. Garber, B. Ehresmann, C. Ehresmann, S. Nikonov, Crystal structure of the S15-rRNA complex, Nature Structural Biology 7 (4) (2000) 273–277. doi:10.1038/74028.
URL http://dx.doi.org/10.1038/74028

[6] R. T. Batey, R. P. Rambo, L. Lucast, B. Rha, J. A. Doudna, Crystal structure of the ribonucleoprotein core of the signal recognition particle, Science 287 (5456) (2000) 1232–1239. doi:10.1126/science.287.5456.1232.
URL http://dx.doi.org/10.1126/science.287.5456.1232

[7] I. Vidovic, S. Nottrott, K. Hartmuth, R. Lührmann, R. Ficner, Crystal structure of the spliceosomal 15.5kD protein bound to a U4 snRNA fragment, Molecular Cell 6 (6) (2000) 1331–1342. doi:10.1016/s1097-2765(00)00131-3.
URL http://dx.doi.org/10.1016/S1097-2765(00)00131-3

[8] H. A. Lewis, K. Musunuru, K. B. Jensen, C. Edo, H. Chen, R. B. Darnell, S. K. Burley, Sequence-specific RNA binding by a nova KH domain, Cell 100 (3) (2000) 323–332. doi:10.1016/s0092-8674(00)80668-6.
URL http://dx.doi.org/10.1016/S0092-8674(00)80668-6

[9] E. Ennifar, A. Nikulin, S. Tishchenko, A. Serganov, N. Nevskaya, M. Garber, B. Ehresmann, C. Ehresmann, S. Nikonov, P. Dumas, The crystal structure of UUCG tetraloop, Journal of Molecular Biology 304 (1) (2000) 35–42. doi:10.1006/jmbi.2000.4204.
URL http://dx.doi.org/10.1006/jmbi.2000.4204

[10] G. L. Conn, A. G. Gittis, E. E. Lattman, V. K. Misra, D. E. Draper, A compact RNA tertiary structure contains a buried backbone-K+ complex, Journal of Molecular Biology 318 (4) (2002) 963–973. doi:10.1016/s0022-2836(02)00147-x.
URL http://dx.doi.org/10.1016/S0022-2836(02)00147-X

[11] S. Tishchenko, A. Nikulin, N. Fomenkova, N. Nevskaya, O. Nikonov, P. Dumas, H. Moine, B. Ehresmann, C. Ehresmann, W. Piendl, V. Lamzin, M. Garber, S. Nikonov, Detailed analysis of RNA-protein interactions within the ribosomal protein S8-rRNA complex from the archaeon methanococcus jannaschii, Journal of Molecular Biology 311 (2) (2001) 311–324. doi:10.1006/jmbi.2001.4877.
URL http://dx.doi.org/10.1006/jmbi.2001.4877

[12] X. Yang, T. Gérczei, L. Glover, C. C. Correll, Crystal structures of restrictocin-inhibitor complexes with implications for RNA recognition and base flipping, Nature Structural Biology 8 (11) (2001) 968–973. doi:10.1038/nsb1101-968.
URL http://dx.doi.org/10.1038/nsb1101-968

[13] K. Wild, I. Sinning, S. Cusack, Crystal structure of an early protein-RNA assembly complex of the signal recognition particle, Science 294 (5542) (2001) 598–601. doi:10.1126/science.1063839.
URL http://dx.doi.org/10.1126/science.1063839

[14] C. Hoang, A. R. Ferré-D'Amaré, Cocrystal structure of a tRNA ψ55 pseudouridine synthase, Cell 107 (7) (2001) 929–939. doi:10.1016/s0092-8674(01)00618-3.
URL http://dx.doi.org/10.1016/S0092-8674(01)00618-3

[15] S. V. Revtovich, A. D. Nikulin, S. V. Nikonov, Role of N-terminal helix in interaction of ribosomal protein S15 with 16S rRNA, Biochemistry (Moscow) 69 (12) (2004) 1319–1323. doi:10.1007/s10541-005-0076-5.
URL http://dx.doi.org/10.1007/s10541-005-0076-5

[16] B. T. Wimberly, R. Guymon, J. P. McCutcheon, S. W. White, V. Ramakrishnan, A detailed view of a ribosomal active site, Cell 97 (4) (1999) 491–502. doi:10.1016/s0092-8674(00)80759-x.
URL http://dx.doi.org/10.1016/S0092-8674(00)80759-X

[17] A. Nikulin, I. Eliseikina, S. Tishchenko, N. Nevskaya, N. Davydova, O. Platonova, W. Piendl, M. Selmer, A. Liljas, D. Drygin, R. Zimmermann, M. Garber, S. Nikonov, Structure of the L1 protuberance in the ribosome, Nature Structural Biology 10 (2) (2003) 104–108. doi:10.1038/nsb886.
URL http://dx.doi.org/10.1038/nsb886

[18] D.-B. Huang, D. Vu, L. A. Cassiday, J. M. Zimmerman, L. J. Maher, G. Ghosh, Crystal structure of nf-κb $(p50)_2$ complexed to a high-affinity RNA aptamer, Proceedings of the National Academy of Sciences 100 (16) (2003) 9268–9273. doi:10.1073/pnas.1632011100.
URL http://dx.doi.org/10.1073/pnas.1632011100

[19] G. L. Conn, D. E. Draper, E. E. Lattman, A. G. Gittis, Crystal structure of a conserved ribosomal protein-RNA complex, Science 284 (5417) (1999) 1171–1174. doi:10.1126/science.284.5417.1171.
URL http://dx.doi.org/10.1126/science.284.5417.1171

[20] K. Ye, L. Malinina, D. J. Patel, Recognition of small interfering RNA by a viral suppressor of RNA silencing, Nature 426 (6968) (2003) 874–878. doi:10.1038/nature02213.
URL http://dx.doi.org/10.1038/nature02213

[21] T. Moore, Y. Zhang, M. O. Fenley, H. Li, Molecular basis of box C/D RNA-protein interactions, Structure 12 (5) (2004) 807–818. doi:10.1016/j.str.2004.02.033.
URL http://dx.doi.org/10.1016/j.str.2004.02.033

[22] H. J. MERIANOS, J. WANG, P. B. MOORE, The structure of a ribosomal protein S8/spc operon mRNA complex, RNA 10 (6) (2004) 954–964. doi:10.1261/rna.7030704.
URL http://dx.doi.org/10.1261/rna.7030704

[23] C. Oubridge, N. Ito, P. R. Evans, C.-H. Teo, K. Nagai, Crystal structure at 1.92 å resolution of the RNA-binding domain of the U1A spliceosomal protein complexed with an RNA hairpin, Nature 372 (6505) (1994) 432–438. doi:10.1038/372432a0.
URL http://dx.doi.org/10.1038/372432a0

[24] M. S. Dunstan, D. GuhaThakurta, D. E. Draper, G. L. Conn, Co-evolution of protein and RNA structures within a highly conserved ribosomal domain, Chemistry and Biology 12 (2) (2005) 201–206. doi:10.1016/j.chembiol.2004.11.019.
URL http://dx.doi.org/10.1016/j.chembiol.2004.11.019

[25] K. Phannachet, Conformational change of pseudouridine 55 synthase upon its association with RNA substrate, Nucleic Acids Research 32 (4) (2004) 1422–1429. doi:10.1093/nar/gkh287.
URL http://dx.doi.org/10.1093/nar/gkh287

[26] M. Teplova, Y.-R. Yuan, A. T. Phan, L. Malinina, S. Ilin, A. Teplov, D. J. Patel, Structural basis for recognition and sequestration of $UUU_{OH}3'$ temini of nascent RNA polymerase III transcripts by La, a rheumatic disease autoantigen, Molecular Cell 21 (1) (2006) 75–85. doi:10.1016/j.molcel.2005.10.027.
URL http://dx.doi.org/10.1016/j.molcel.2005.10.027

[27] C. Hoang, C. S. Hamilton, E. G.Mueller, A. R. Ferré-D'Amaré, Precursor complex structure of pseudouridine synthase trub suggests coupling of active site perturbations to an RNA-sequestering peripheral protein domain, Protein Science 14 (8) (2005) 2201–2206. doi:10.1110/ps.051493605.
URL http://dx.doi.org/10.1110/ps.051493605

[28] W. T. Horn, K. Tars, E. Grahn, C. Helgstrand, A. J. Baron, H. Lago, C. J. Adams, D. S. Peabody, S. E. Phillips, N. J. Stonehouse, L. Liljas, P. G. Stockley, Structural basis of RNA binding discrimination between bacteriophages qβ and MS2, Structure 14 (3) (2006) 487–495. doi:10.1016/j.str.2005.12.006.
URL http://dx.doi.org/10.1016/j.str.2005.12.006

[29] M. Teplova, L. Malinina, J. Darnell, J. Song, M. Lu, R. Abagyan, K. Musunuru, A. Teplov, S. Burley, R. Darnell, D. Patel, Protein-RNA and protein-protein recognition by dual KH1/2 domains of the neuronal splicing factor nova-1, Structure 19 (7) (2011) 930–944.

doi:10.1016/j.str.2011.05.002.
URL http://dx.doi.org/10.1016/j.str.2011.05.002

[30] K. Tomita, R. Ishitani, S. Fukai, O. Nureki, Complete crystallographic analysis of the dynamics of CCA sequence addition, Nature 443 (7114) (2006) 956–960. doi:10.1038/nature05204.
URL http://dx.doi.org/10.1038/nature05204

[31] T. Aviv, Z. Lin, G. Ben-Ari, C. A. Smibert, F. Sicheri, Sequence-specific recognition of RNA hairpins by the SAM domain of Vts1p, Nature Structural and Molecular Biology 13 (2) (2006) 168–176. doi:10.1038/nsmb1053.
URL http://dx.doi.org/10.1038/nsmb1053

[32] L. Li, K. Ye, Crystal structure of an H/ACA box ribonucleoprotein particle, Nature 443 (7109) (2006) 302–307. doi:10.1038/nature05151.
URL http://dx.doi.org/10.1038/nature05151

[33] S. Tishchenko, E. Nikonova, A. Nikulin, N. Nevskaya, S. Volchkov, W. Piendl, M. Garber, S. Nikonov, Structure of ribosomal protein L1-mRNA complex at 2.1å resolution: common features of crystal packing of L1-RNA complexes, Acta Crystallographica Section D Biological Crystallography 62 (12) (2006) 1545–1554. doi:10.1107/s0907444906041655.
URL http://dx.doi.org/10.1107/S0907444906041655

[34] N. Soler, D. Fourmy, S. Yoshizawa, Structural insight into a molecular switch in tandem winged-helix motifs from elongation factor SelB, Journal of Molecular Biology 370 (4) (2007) 728–741. doi:10.1016/j.jmb.2007.05.001.
URL http://dx.doi.org/10.1016/j.jmb.2007.05.001

[35] T. R. Blower, X. Y. Pei, F. L. Short, P. C. Fineran, D. P. Humphreys, B. F. Luisi, G. P. C. Salmond, A processed noncoding RNA regulates an altruistic bacterial antiviral system, Nature Structural and Molecular Biology 18 (2) (2011) 185–190. doi:10.1038/nsmb.1981.
URL http://dx.doi.org/10.1038/NSMB.1981

[36] D. G. Sashital, M. Jinek, J. A. Doudna, An RNA-induced conformational change required for CRISPR RNA cleavage by the endoribonuclease Cse3, Nature Structural and Molecular Biology 18 (6) (2011) 680–687. doi:10.1038/nsmb.2043.
URL http://dx.doi.org/10.1038/NSMB.2043

[37] S. Goto-Ito, T. Ito, M. Kuratani, Y. Bessho, S. Yokoyama, Tertiary structure checkpoint at anticodon loop modification in tRNA functional maturation, Nat. Struct. Mol. Biol. 16 (10) (2009) 1109–1115. doi:10.1038/nsmb.1653.
URL http://dx.doi.org/10.1038/nsmb.1653

[38] T. Osawa, S. Kimura, N. Terasaka, H. Inanaga, T. Suzuki, T. Numata, Structural basis of tRNA agmatinylation essential for AUA codon decoding, Nat. Struct. Mol. Biol. 18 (11) (2011) 1275–1280. doi:10.1038/nsmb.2144.
URL http://dx.doi.org/10.1038/nsmb.2144

[39] A. Alian, T. T. Lee, S. L. Griner, R. M. Stroud, J. Finer-Moore, Structure of a TrmA-RNA complex: A consensus RNA fold contributes to substrate selectivity and catalysis in m^5U methyltransferases, Proceedings of the National Academy of Sciences 105 (19) (2008) 6876–6881. doi:10.1073/pnas.0802247105.
URL http://dx.doi.org/10.1073/pnas.0802247105

[40] A. Perederina, O. Esakova, C. Quan, E. Khanova, A. S. Krasilnikov, Eukaryotic ribonucleases P/MRP: the crystal structure of the P3 domain, The EMBO Journal 29 (4) (2010) 761–769. doi:10.1038/emboj.2009.396.
URL http://dx.doi.org/10.1038/emboj.2009.396

[41] S. R. Thomas, C. A. Keller, A. Szyk, J. R. Cannon, N. A. LaRonde-LeBlanc, Structural insight into the functional mechanism of Nep1/Emg1 N1-specific pseudouridine methyltransferase in ribosome biogenesis, Nucleic Acids Res. 39 (6) (2010) 2445–2457. doi:10.1093/nar/gkq1131.
URL http://dx.doi.org/10.1093/nar/gkq1131

[42] S. Tishchenko, A. Gabdulkhakov, N. Nevskaya, A. Sarskikh, O. Kostareva, E. Nikonova, A. Sycheva, S. Moshkovskii, M. Garber, S. Nikonov, High-resolution crystal structure of the isolated ribosomal L1 stalk, Acta Crystallographica Section D Biological Crystallography 68 (8) (2012) 1051–1057. doi:10.1107/s0907444912020136.
URL http://dx.doi.org/10.1107/S0907444912020136

[43] R. E. Haurwitz, S. H. Sternberg, J. A. Doudna, Csy4 relies on an unusual catalytic dyad to position and cleave CRISPR RNA, The EMBO Journal 31 (12) (2012) 2824–2832. doi:10.1038/emboj.2012.107.
URL http://dx.doi.org/10.1038/EMBOJ.2012.107

[44] L. Huang, D. M. Lilley, The molecular recognition of kink-turn structure by the L7Ae class of proteins, RNA 19 (12) (2013) 1703–1710. doi:10.1261/rna.041517.113.
URL http://dx.doi.org/10.1261/RNA.041517.113

[45] O. Niewoehner, M. Jinek, J. A. Doudna, Evolution of CRISPR RNA recognition and processing by Cas6 endonucleases, Nucleic Acids Research 42 (2) (2013) 1341–1353. doi:10.1093/nar/gkt922.
URL http://dx.doi.org/10.1093/NAR/GKT922

[46] O. Pellegrini, I. Li de la Sierra-Gallay, J. Piton, L. Gilet, C. Condon, Activation of tRNA maturation by downstream uracil residues in b. subtilis, Structure 20 (10) (2012) 1769–1777. doi:10.1016/j.str.2012.08.002.
URL http://dx.doi.org/10.1016/j.str.2012.08.002

[47] J. Rumnieks, K. Tars, Crystal structure of the bacteriophage Qβ coat protein in complex with the RNA operator of the replicase gene, Journal of Molecular Biology 426 (5) (2014) 1039–1049. doi:10.1016/j.jmb.2013.08.025.
URL http://dx.doi.org/10.1016/j.jmb.2013.08.025

[48] N. Czudnochowski, G. W. Ashley, D. V. Santi, A. Alian, J. Finer-Moore, R. M. Stroud, The mechanism of pseudouridine synthases from a covalent complex with RNA, and alternate specificity for U2605 versus U2604 between close homologs, Nucleic Acids Research 42 (3) (2013) 2037–2048. doi:10.1093/nar/gkt1050.
URL http://dx.doi.org/10.1093/nar/gkt1050

[49] M. Davlieva, J. Donarski, J. Wang, Y. Shamoo, E. P. Nikonowicz, Structure analysis of free and bound states of an RNA aptamer against ribosomal protein S8 from bacillus anthracis, Nucleic Acids Research 42 (16) (2014) 10795–10808. doi:10.1093/nar/gku743.
URL http://dx.doi.org/10.1093/nar/gku743

[50] D. Tan, M. Zhou, M. Kiledjian, L. Tong, The ROQ domain of roquin recognizes mRNA constitutive-decay element and double-stranded RNA, Nature Structural and Molecular Biology 21 (8) (2014) 679–685. doi:10.1038/nsmb.2857.
URL http://dx.doi.org/10.1038/nsmb.2857

[51] D. Oberthür, J. Achenbach, A. Gabdulkhakov, K. Buchner, C. Maasch, S. Falke, D. Rehders, S. Klussmann, C. Betzel, Crystal structure of a

mirror-image l-RNA aptamer (spiegelmer) in complex with the natural L-protein target CCL2, Nature Communications 6 (1) (apr 2015). doi:10.1038/ncomms7923.
URL http://dx.doi.org/10.1038/ncomms7923

[52] J. Zhang, D. Tan, E. F. DeRose, L. Perera, Z. Dominski, W. F. Marzluff, L. Tong, T. M. T. Hall, Molecular mechanisms for the regulation of histone mRNA stem-loop-binding protein by phosphorylation, Proceedings of the National Academy of Sciences 111 (29) (jul 2014). doi:10.1073/pnas.1406381111.
URL http://dx.doi.org/10.1073/pnas.1406381111

[53] R. T. Byrne, H. T. Jenkins, D. T. Peters, F. Whelan, J. Stowell, N. Aziz, P. Kasatsky, M. V. Rodnina, E. V. Koonin, A. L. Konevega, A. A. Antson, Major reorientation of tRNA substrates defines specificity of dihydrouridine synthases, Proc. Natl. Acad. Sci. 112 (19) (2015) 6033–6037. doi:10.1073/pnas.1500161112.
URL http://dx.doi.org/10.1073/pnas.1500161112

[54] T. Ito, I. Masuda, K.-i. Yoshida, S. Goto-Ito, S.-i. Sekine, S. W. Suh, Y.-M. Hou, S. Yokoyama, Structural basis for methyl-donor-dependent and sequence-specific binding to tRNA substrates by knotted methyltransferase TrmD, Proceedings of the National Academy of Sciences 112 (31) (jul 2015). doi:10.1073/pnas.1422981112.
URL http://dx.doi.org/10.1073/pnas.1422981112

[55] S. Sakurai, U. Ohto, T. Shimizu, Structure of human roquin-2 and its complex with constitutive-decay element RNA, Acta Crystallographica Section F Structural Biology Communications 71 (8) (2015) 1048–1054. doi:10.1107/s2053230x15011887.
URL http://dx.doi.org/10.1107/S2053230X15011887

[56] V. Ahl, H. Keller, S. Schmidt, O. Weichenrieder, Retrotransposition and crystal structure of an alu RNP in the ribosome-stalling conformation, Molecular Cell 60 (5) (2015) 715–727. doi:10.1016/j.molcel.2015.10.003.
URL http://dx.doi.org/10.1016/J.MOLCEL.2015.10.003

[57] N. Vasilyev, A. Polonskaia, J. C. Darnell, R. B. Darnell, D. J. Patel, A. Serganov, Crystal structure reveals specific recognition of a G-quadruplex RNA by a β-turn in the RGG motif of FMRP, Proc. Natl. Acad. Sci. 112 (39) (sep 2015). doi:10.1073/pnas.1515737112.
URL http://dx.doi.org/10.1073/pnas.1515737112

[58] N. D. Abeydeera, M. Egli, N. Cox, K. Mercier, J. N. Conde, P. S. Pallan, D. M. Mizurini, M. Sierant, F.-E. Hibti, T. Hassell, T. Wang, F.-W. Liu, H.-M. Liu, C. Martinez, A. K. Sood, T. P. Lybrand, C. Frydman, R. Q. Monteiro, R. H. Gomer, B. Nawrot, X. Yang, Evoking picomolar binding in rna by a single phosphorodithioate linkage, Nucleic Acids Res. 44 (17) (2016) 8052–8064. doi:10.1093/nar/gkw725.
URL http://dx.doi.org/10.1093/nar/gkw725

[59] R. Janowski, G. A. Heinz, A. Schlundt, N. Wommelsdorf, S. Brenner, A. R. Gruber, M. Blank, T. Buch, R. Buhmann, M. Zavolan, D. Niessing, V. Heissmeyer, M. Sattler, Roquin recognizes a non-canonical hexaloop structure in the 3′-UTR of Ox40, Nature Communications 7 (1) (mar 2016). doi:10.1038/ncomms11032.
URL http://dx.doi.org/10.1038/ncomms11032

[60] T. Suzuki, C. Miller, L.-T. Guo, J. M. L. Ho, D. I. Bryson, Y.-S. Wang, D. R. Liu, D. Söll, Crystal structures reveal an elusive functional domain of pyrrolysyl-tRNA synthetase, Nature Chemical Biology 13 (12) (2017) 1261–1266. doi:10.1038/nchembio.2497.
URL http://dx.doi.org/10.1038/nchembio.2497

[61] R. Gunaratne, S. Kumar, J. W. Frederiksen, S. Stayrook, J. L. Lohrmann, K. Perry, K. M. Bompiani, C. V. Chabata, N. K. Thalji, M. D. Ho, G. Arepally, R. M. Camire, S. Krishnaswamy, B. A. Sullenger, Combination of aptamer and drug for reversible anticoagulation in cardiopulmonary bypass, Nature Biotechnology 36 (7) (2018) 606–613. doi:10.1038/nbt.4153.
URL http://dx.doi.org/10.1038/nbt.4153

[62] B. Wu, S. Su, D. P. Patil, H. Liu, J. Gan, S. R. Jaffrey, J. Ma, Molecular basis for the specific and multivariant recognitions of RNA substrates by human hnrnp A2/B1, Nature Communications 9 (1) (jan 2018). doi:10.1038/s41467-017-02770-z.
URL http://dx.doi.org/10.1038/s41467-017-02770-z

[63] L. Yang, C. Wang, F. Li, J. Zhang, A. Nayab, J. Wu, Y. Shi, Q. Gong, The human RNA-binding protein and E3 ligase MEX-3C binds the MEX-3-recognition element (MRE) motif with high affinity, J. Biol. Chem. 292 (39) (2017) 16221–16234. doi:10.1074/jbc.m117.797746.
URL http://dx.doi.org/10.1074/jbc.M117.797746

[64] Y. Jiang, H. Yu, F. Li, L. Cheng, L. Zhu, Y. Shi, Q. Gong, Unveiling the structural features that determine the dual methyltransferase activities of streptococcus pneumoniae RlmCD, PLoS Pathog. 14 (11) (2018) e1007379. doi:10.1371/journal.ppat.1007379.
URL http://dx.doi.org/10.1371/journal.ppat.1007379

[65] H. Ryu, T. L. Grove, S. C. Almo, J. Kim, Identification of a novel tRNA wobble uridine modifying activity in the biosynthesis of 5-methoxyuridine, Nucleic Acids Res. 46 (17) (2018) 9160–9169. doi:10.1093/nar/gky592.
URL http://dx.doi.org/10.1093/nar/gky592

[66] X. Liu, S. Shen, P. Wu, F. Li, X. Liu, C. Wang, Q. Gong, J. Wu, X. Yao, H. Zhang, Y. Shi, Structural insights into dimethylation of 12S rRNA by TFB1M: indispensable role in translation of mitochondrial genes and mitochondrial function, Nucleic Acids Res. 47 (14) (2019) 7648–7665. doi:10.1093/nar/gkz505.
URL http://dx.doi.org/10.1093/nar/gkz505

[67] A. D. Dearborn, E. Eren, N. R. Watts, I. W. Palmer, J. D. Kaufman, A. C. Steven, P. T. Wingfield, Structure of an RNA aptamer that can inhibit HIV-1 by blocking rev-cognate RNA (rre) binding and rev-rev association, Structure 26 (9) (2018) 1187–1195.e4. doi:10.1016/j.str.2018.06.001.
URL http://dx.doi.org/10.1016/j.str.2018.06.001

[68] I. A. Belashov, D. W. Crawford, C. E. Cavender, P. Dai, P. C. Beardslee, D. H. Mathews, B. L. Pentelute, B. R. McNaughton, J. E. Wedekind, Structure of HIV TAR in complex with a lab-evolved RRM provides insight into duplex RNA recognition and synthesis of a constrained peptide that impairs transcription, Nucleic Acids Research 46 (13) (2018) 6401–6415. doi:10.1093/nar/gky529.
URL http://dx.doi.org/10.1093/nar/gky529

[69] Y. Yang, C. D. Eichhorn, Y. Wang, D. Cascio, J. Feigon, Structural basis of 7SK RNA 5′-γ-phosphate methylation and retention by MePCE, Nat. Chem. Biol. 15 (2) (2018) 132–140. doi:10.1038/s41589-018-0188-z.
URL http://dx.doi.org/10.1038/s41589-018-0188-z

[70] K. A. Doxtader, P. Wang, A. M. Scarborough, D. Seo, N. K. Conrad, Y. Nam, Structural basis for regulation of METTL16, an S-adenosylmethionine homeostasis factor, Molecular Cell 71 (6) (2018)

1001–1011.e4. doi:10.1016/j.molcel.2018.07.025.
URL http://dx.doi.org/10.1016/j.molcel.2018.07.025

[71] G. Weber, G. T. DeKoster, N. Holton, K. B. Hall, M. C. Wahl, Molecular principles underlying dual RNA specificity in the drosophila SNF protein, Nature Communications 9 (1) (jun 2018). doi:10.1038/s41467-018-04561-6.
URL http://dx.doi.org/10.1038/s41467-018-04561-6

[72] P. Kumari, F. Aeschimann, D. Gaidatzis, J. J. Keusch, P. Ghosh, A. Neagu, K. Pachulska-Wieczorek, J. M. Bujnicki, H. Gut, H. Großhans, R. Ciosk, Evolutionary plasticity of the NHL domain underlies distinct solutions to RNA recognition, Nature Communications 9 (1) (apr 2018). doi:10.1038/s41467-018-03920-7.
URL http://dx.doi.org/10.1038/s41467-018-03920-7

[73] A. Özcan, P. Pausch, A. Linden, A. Wulf, K. Schühle, J. Heider, H. Urlaub, T. Heimerl, G. Bange, L. Randau, Type IV CRISPR RNA processing and effector complex formation in aromatoleum aromaticum, Nature Microbiology 4 (1) (2018) 89–96. doi:10.1038/s41564-018-0274-8.
URL http://dx.doi.org/10.1038/s41564-018-0274-8

[74] K. Qian, M. Li, J. Wang, M. Zhang, M. Wang, Structural basis for mRNA recognition by human RBM38, Biochemical Journal 477 (1) (2020) 161–172. doi:10.1042/bcj20190652.
URL http://dx.doi.org/10.1042/BCJ20190652

[75] A. Sun, C. Gasser, F. Li, H. Chen, S. Mair, O. Krasheninina, R. Micura, A. Ren, SAM-VI riboswitch structure and signature for ligand discrimination, Nat. Commun. 10 (1) (dec 2019). doi:10.1038/s41467-019-13600-9.
URL http://dx.doi.org/10.1038/s41467-019-13600-9

[76] E. J. Montemayor, J. M. Virta, S. M. Hayes, Y. Nomura, D. A. Brow, S. E. Butcher, Molecular basis for the distinct cellular functions of the Lsm1-7 and Lsm2-8 complexes, RNA 26 (10) (2020) 1400–1413. doi:10.1261/rna.075879.120.
URL http://dx.doi.org/10.1261/rna.075879.120

[77] A. Wacquiez, F. Coste, E. Kut, V. Gaudon, S. Trapp, B. Castaing, D. Marc, Structure and sequence determinants governing the interactions of RNAs with influenza A virus non-structural protein NS1,

Viruses 12 (9) (2020) 947. doi:10.3390/v12090947.
URL http://dx.doi.org/10.3390/v12090947

[78] F. C. Grau, J. Jaeger, F. Groher, B. Suess, Y. A. Muller, The complex formed between a synthetic RNA aptamer and the transcription repressor TetR is a structural and functional twin of the operator DNA-TetR regulator complex, Nucleic Acids Research 48 (6) (2020) 3366–3378. doi:10.1093/nar/gkaa083.
URL http://dx.doi.org/10.1093/nar/gkaa083

[79] O. Binas, J.-N. Tants, S. A. Peter, R. Janowski, E. Davydova, J. Braun, D. Niessing, H. Schwalbe, J. Weigand, A. Schlundt, Structural basis for the recognition of transiently structured AU-rich elements by roquin, Nucleic Acids Research (jun 2020). doi:10.1093/nar/gkaa465.
URL http://dx.doi.org/10.1093/nar/gkaa465

[80] S. S. Chavali, S. M. Mali, J. L. Jenkins, R. Fasan, J. E. Wedekind, Co-crystal structures of HIV TAR RNA bound to lab-evolved proteins show key roles for arginine relevant to the design of cyclic peptide TAR inhibitors, Journal of Biological Chemistry 295 (49) (2020) 16470–16486. doi:10.1074/jbc.ra120.015444.
URL http://dx.doi.org/10.1074/jbc.RA120.015444

[81] S. K. Naineni, J. Liang, K. Hull, R. Cencic, M. Zhu, P. Northcote, P. Teesdale-Spittle, D. Romo, B. Nagar, J. Pelletier, Functional mimicry revealed by the crystal structure of an eIF4A:RNA complex bound to the interfacial inhibitor, desmethyl pateamine A, Cell Chem. Biol. 28 (6) (2021) 825–834.e6. doi:10.1016/j.chembiol.2020.12.006.
URL http://dx.doi.org/10.1016/j.chembiol.2020.12.006

[82] V. Andric, A. Nevers, D. Hazra, S. Auxilien, A. Menant, M. Graille, B. Palancade, M. Rougemaille, A scaffold lncRNA shapes the mitosis to meiosis switch, Nature Communications 12 (1) (feb 2021). doi:10.1038/s41467-021-21032-7.
URL http://dx.doi.org/10.1038/s41467-021-21032-7

[83] Y. Yang, K. A. Harris, D. L. Widner, R. R. Breaker, Structure of a bacterial OapB protein with its OLE RNA target gives insights into the architecture of the OLE ribonucleoprotein complex, Proc. Natl. Acad. Sci. 118 (9) (feb 2021). doi:10.1073/pnas.2020393118.
URL http://dx.doi.org/10.1073/pnas.2020393118

[84] O. A. Esakova, T. L. Grove, N. H. Yennawar, A. J. Arcinas, B. Wang, C. Krebs, S. C. Almo, S. J. Booker, Structural basis for tRNA methylthiolation by the radical SAM enzyme MiaB, Nature 597 (7877) (2021) 566–570. doi:10.1038/s41586-021-03904-6.
URL http://dx.doi.org/10.1038/s41586-021-03904-6

[85] S. Höfler, P. Lukat, W. Blankenfeldt, T. Carlomagno, Eukaryotic box C/D methylation machinery has two non-symmetric protein assembly sites, Scientific Reports 11 (1) (sep 2021). doi:10.1038/s41598-021-97030-y.
URL http://dx.doi.org/10.1038/s41598-021-97030-y

[86] T. de Vries, W. Martelly, S. Campagne, K. Sabath, C. P. Sarnowski, J. Wong, A. Leitner, S. Jonas, S. Sharma, F. H.-T. Allain, Sequence-specific RNA recognition by an RGG motif connects U1 and U2 snRNP for spliceosome assembly, Proceedings of the National Academy of Sciences 119 (6) (jan 2022). doi:10.1073/pnas.2114092119.
URL http://dx.doi.org/10.1073/pnas.2114092119

[87] M. Zhang, R. Peng, Q. Peng, S. Liu, Z. Li, Y. Zhang, H. Song, J. Yang, X. Xing, P. Wang, J. Qi, G. F. Gao, Mechanistic insights into dna binding and cleavage by a compact type I-F CRISPR-Cas system in bacteriophage, Proceedings of the National Academy of Sciences 120 (18) (apr 2023). doi:10.1073/pnas.2215098120.
URL http://dx.doi.org/10.1073/pnas.2215098120

[88] H. Betat, M. Mörl, The CCA-adding enzyme: A central scrutinizer in tRNA quality control, BioEssays 37 (9) (2015) 975–982. doi:10.1002/bies.201500043.
URL http://dx.doi.org/10.1002/bies.201500043

[89] G. Bolcato, E. Heid, J. Boström, On the value of using 3D shape and electrostatic similarities in deep generative methods, J. Chem. Inf. Model. 62 (6) (2022) 1388–1398. doi:10.1021/acs.jcim.1c01535.
URL http://dx.doi.org/10.1021/acs.jcim.1c01535

[90] D. A. Case, H. M. Aktulga, K. Belfon, I. Y. Ben-Shalom, J. T. Berryman, S. R. Brozell, D. S. Cerutti, T. E. Cheatham III, G. A. Cisneros, V. W. D. Cruzeiro, T. A. Darden, N. Forouzesh, G. Giambaşu, T. Giese, M. K. Gilson, H. Gohlke, A. W. Goetz, J. Harris, S. Izadi, S. A. Izmailov, K. Kasavajhala, M. C. Kaymak, E. King, A. Kovalenko, T. Kurtzman, T. S. Lee, P. Li, C. Lin, J. Liu, T. Luchko, R. Luo, M. Machado, V. Man,

M. Manathunga, K. M. Merz, Y. Miao, O. Mikhailovskii, G. Monard, H. Nguyen, K. A. O'Hearn, A. Onufriev, F. Pan, S. Pantano, R. Qi, A. Rahnamoun, D. R. Roe, A. Roitberg, C. Sagui, S. Schott-Verdugo, A. Shajan, J. Shen, C. L. Simmerling, N. R. Skrynnikov, J. Smith, J. Swails, R. C. Walker, J. Wang, J. Wang, H. Wei, X. Wu, Y. Wu, Y. Xiong, Y. Xue, D. M. York, S. Zhao, Q. Zhu, P. A. Kollman, Ambertools23 (2023).
URL ambermd.org

[91] J. Degen, C. Wegscheid-Gerlach, A. Zaliani, M. Rarey, On the art of compiling and using "drug-like" chemical fragment spaces, ChemMedChem 3 (10) (2008) 1503–1507. doi:10.1002/cmdc.200800178.
URL http://dx.doi.org/10.1002/cmdc.200800178
